# DNA-based artificial molecular signaling system that mimics basic elements of reception and response

**DOI:** 10.1038/s41467-020-14739-6

**Published:** 2020-02-20

**Authors:** Ruizi Peng, Liujun Xu, Huijing Wang, Yifan Lyu, Dan Wang, Cheng Bi, Cheng Cui, Chunhai Fan, Qiaoling Liu, Xiaobing Zhang, Weihong Tan

**Affiliations:** 1 Molecular Science and Biomedicine Laboratory (MBL), State Key Laboratory of Chemo/Bio-Sensing and Chemometrics, College of Chemistry and Chemical Engineering, College of Biology, and Aptamer Engineering Center of Hunan Province, Hunan University, Changsha, Hunan 410082 People’s Republic of China; 2 Institute of Molecular Medicine (IMM), State Key Laboratory of Oncogenes and Related Genes Renji Hospital, Shanghai Jiao Tong University School of Medicine, and College of Chemistry and Chemical Engineering, Shanghai Jiao Tong University, Shanghai, 200240 China; 3 Institute of Cancer and Basic Medicine (IBMC), Chinese Academy of Sciences, The Cancer Hospital of the University of Chinese Academy of Sciences, Hangzhou, Zhejiang 310022 China; 4 Foundation for Applied Molecular Evolution, 13709 Progress Boulevard, Alachua, FL 32615 USA

**Keywords:** Synthetic biology, Chemical tools, Networks and systems biology

## Abstract

In order to maintain tissue homeostasis, cells communicate with the outside environment by receiving molecular signals, transmitting them, and responding accordingly with signaling pathways. Thus, one key challenge in engineering molecular signaling systems involves the design and construction of different modules into a rationally integrated system that mimics the cascade of molecular events. Herein, we rationally design a DNA-based artificial molecular signaling system that uses the confined microenvironment of a giant vesicle, derived from a living cell. This system consists of two main components. First, we build an adenosine triphosphate (ATP)-driven DNA nanogatekeeper. Second, we encapsulate a signaling network in the biomimetic vesicle, consisting of distinct modules, able to sequentially initiate a series of downstream reactions playing the roles of reception, transduction and response. Operationally, in the presence of ATP, nanogatekeeper switches from the closed to open state. The open state then triggers the sequential activation of confined downstream signaling modules.

## Abstract (web summary)

Cells communicate with the outside world to maintain homeostasis. Here the authors design a synthetic biology DNA-based signalling system AMSsys that responds to the presence of ATP.

## Introduction

Cell signaling systems consist of interwoven molecular reaction networks in confined compartments with the capacity to process information from environmental stimuli^1,2^. Inspired by such intracellular information processing, researchers have been able to develop sophisticated artificial molecular signaling systems, including neural network computation^3^, adaptive immune response circuits^4^, and molecular cascades^5^. Using DNA as basic building blocks, scientists have constructed biomimetic architectures extensively used in research and industry to monitor biological processes^6,7^, perform logic gate operations and cascade reaction networks^8,9^, and develop theranostic nanorobots^10–12^.

Biomimetic DNA nanostructures, such as nanopores^13,14^, cytoskeletal filaments^15^, or molecular motors^16,17^_,_ have been employed to make functional modules. Works on biomimetic DNA nanotechnology have so far focused on the fabrication of advanced DNA nanostructures with the goal of developing new building blocks that mimic biological functions in homogeneous solution^18–20^. Focusing on the single cell, intracellular signaling involves an interwoven matrix of molecular reaction networks that process information from environmental stimuli. Importantly, such intricate cell signaling involves membrane receptors at the cell boundary^1,2^. This means that cell signaling mimicry must involve both membrane nanostructures, i.e., protein receptors, as well as an encapsulated signal switching network able to sequentially initiate a series of downstream events to replicate the basic elements of cell signaling: reception, transduction, and response to incoming environmental cues.

Accordingly, we have herein designed and constructed an artificial molecular signaling system (AMSsys), a rationally integrated system able to mimic the cascade of molecular events from signal reception to transduction and, finally, response, all based on an isolated biomimetic giant vesicle. Thus, in response to an outside stimulus, in this case adenosine triphosphate (ATP), a membrane-spanning DNA nanogatekeeper (DNGK), switches from the closed to open state. This, in turn, activates our integrated giant plasma membrane vesicle (GPMV, Supplementary Table 1)-encapsulated signal switching network, consisting of distinct cell-signaling modules, to sequentially accomplish a series of cell-mimicking reactions involving reception, transduction and response. Besides internal response, a feedback pathway returns the on-membrane nanogatekeeper to the closed state. Here, our previously developed GPMVs were derived from detached living cells, providing a cell-mimicking platform with which to manipulate DNA nanostructures, clearly demonstrating the feasibility of engineering a biomimetic membrane compartment^21^. Now, by rationally integrating DNA nanostructure and dynamic cascade networks into this biomimetic GPMV, we demonstrate that an artificial molecular signaling system can, indeed, process environmental cues and react with downstream signaling that mimics real cellular behavior.

**Fig. 1:**
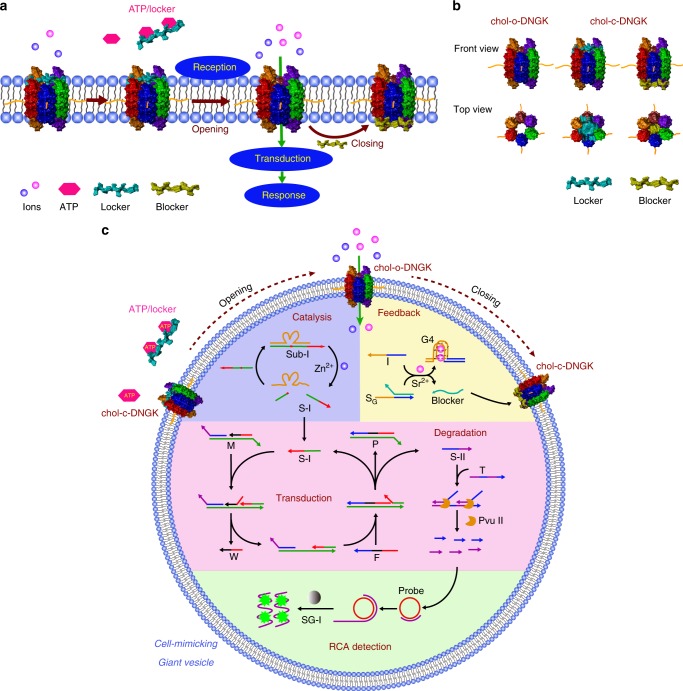
Biomimetic giant vesicle engineered for the construction of an artificial molecular signaling system (AMSsys). **a** The whole system was constructed based on a cell-mimicking giant membrane vesicle. Driven by adenosine triphosphate (ATP), the closed state DNA nanogatekeeper switches to the open state. Then, environmental ions diffuse through the opened channel, sequentially triggering a set of confined downstream cascade reactions, and an additional feedback pathway mediates the switching of nanogatekeeper back to the closed state. This process mimics the fundamental cellular signaling that contains reception, transduction, and response. **b** Schematic illustration of chol-o(open)-DNGK and chol-c(closed)-DNGK. A locker (cyan DNA strand) can plug the nanogatekeeper in the top region; similarly, a blocker (yellow DNA strand) can plug the nanogatekeeper in the bottom region, both locker and blocker can form a closed nanogatekeeper. **c** Modular design of AMSsys in giant membrane vesicles corresponding to **a**, consisting of membrane-spanning nanogatekeeper and the encapsulated signaling network, which is divided into the following modules: ion-mediated catalytic reaction, a DNA circuit signaling transduction, and a target degradation. The rolling circle amplification (RCA) in the end is used to detect the final product.

This system consists of two main components. First, we build an ATP-driven, membrane-spanning DNGK to receive outside stimulus. Second, within the biomimetic GPMV, we further integrate an encapsulated signal switching network that can sequentially initiate a series of downstream reactions that transduce the signal and respond to incoming environmental cues (Fig. 1a). Operationally, after ATP-driven reception for gate-opening, a catalytic reaction initially occurs and triggers a secondary signaling transduction, finally leading to target degradation. A concurrent signal released from the feedback pathway switches nanogatekeeper back to the closed state after cellular response.

## Results

### Design of AMSsys

Ion channels are involved in many cellular processes in which an activated ion channel triggers a cascade of downstream reactions. Inspired by this universal model, we attempted to build an AMSsys based on a cell-mimicking GPMV by integrating a membrane-spanning nanogatekeeper and an encapsulated signaling network.

To create a membrane-spanning nanostructure, cholesterol-labeled nanogatekeeper (chol-DNGK) was constructed for membrane anchoring through hydrophobic cholesterol insertion^22^. Since the bottom region of chol-DNGK contains hydrophobic phosphorothioate groups, such membrane attachment was initially achieved during incubation (chol-o-DNGK in Fig. 1b). To implement the switching property between the open and closed states of the mimetic ion channel, the top region of chol-DNGK is used to bind with the locker, a DNA oligonucleotide, while the bottom region is used to bind with the blocker; either locker or blocker can plug the chol-DNGK (chol-c-DNGK in Fig. 1b). In the presence of ATP, the locker plugged chol-c(closed)-DNGK switches to the open state (chol-o(opened)-DNGK)^23^. Then, environmental ions pass through the opened channel, triggering the confined downstream cascaded reactions that constitute the signaling network. A concurrent feedback signal mediates the switching of open state nanogatekeeper back to the closed state upon successful cellular response. The encapsulated signaling network mimics cellular signaling transduction and response that sequentially includes an ion-mediated catalytic reaction, a DNA circuit signaling transduction, and a target degradation reaction. The degraded DNA segments, as the final product, are detected by rolling circle amplification (RCA), a technique used for sensing single-stranded DNA^24^ (Fig. 1c).

### Construction of an ATP-responsive nanogatekeeper

**Fig. 2:**
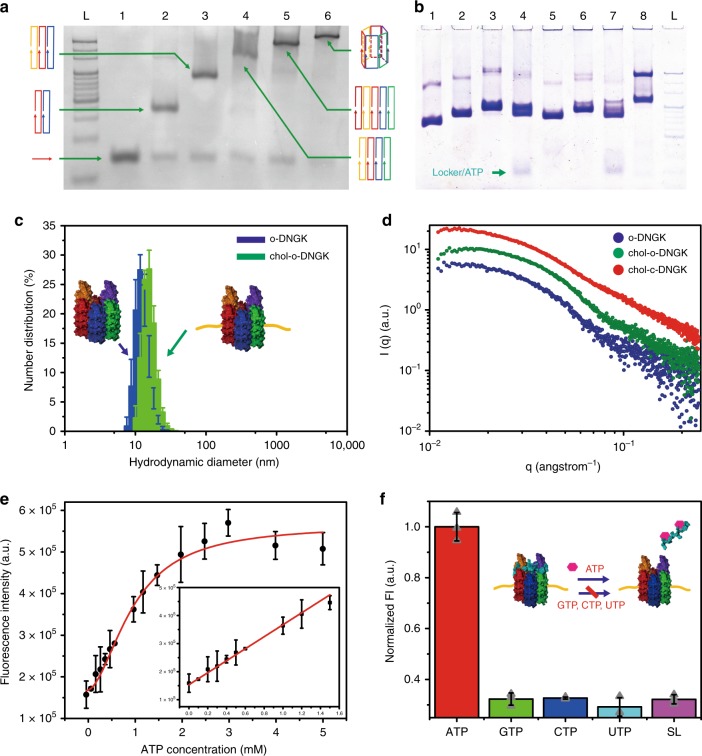
One-pot DNA nanogatekeeper assembly in solution and ATP response. **a** PAGE analysis of stepwise assembly of DNA nanochannel. The bands of DNA nanochannel (lane 6) and assemblies of one to five different component strands (Lanes 1–5, respectively) are shown with high yield. L: 20 bp ladder. **b** Five percent PAGE analysis of ATP response of chol-DNGK. Lane 1: DNA nanochannel; lane 2: o-DNGK; lane 3: c-DNGK; lane 4: c-DNGK with addition of 3 mM ATP; Lane 5: chol-o-DNGK; Lane 6: chol-c-DNGK; Lane 7: chol-c-DNGK with addition of 3 mM ATP; Lane 8: blocker version DNGK with a blocker. Lane L: 20 bp DNA ladder. **c** Dynamic light scattering (DLS) verified the size changes between nanogatekeeper and chol-DNGK. Error bars show the standard deviation of three independent experiments. **d** Comparison of the small-angle X-ray scattering (SAXS) profile from o-DNGK (blue) with scattering profiles of chol-o-DNGK (green) and chol-c-DNGK (red), showing the changes between each group. **e** Fluorescence intensity shows the effect of adding various concentrations of ATP, which causes chol-c-DNGK switch to chol-o-DNGK, and the released locker (fluorophore labeled) enables the “OFF” to “ON” signal readout. **f** Response of c-DNGK to different analogs. The rate of fluorescence enhancement was measured in the presence of 3 mM ATP, guanosine-5’-triphosphate (GTP), cytidine triphosphate (CTP), uridine-5’-triphosphate (UTP) and a scrambled lid (SL) on nanogatekeeper with ATP, respectively. *λ*_ex_ = 492 nm, bandpass = 5 nm. Error bars show the standard deviation of three independent experiments. Source data are provided in the Source Data file.

All DNA nanostructures were self-assembled by programmable annealing. A barrel-like DNA nanochannel was initially constructed based on the 6-helix bundle (a particular motif of cooperative protein folding whereby a protein necessarily gains its native 3-D structure by a series of intramolecular interactions), according to reported studies (Supplementary Figs. 1 and 2, and Supplementary Tables 2–4)^25,26^. Native polyacrylamide gel electrophoresis (PAGE) was employed to analyze the stepwise assembly of the DNA nanochannel (Fig. 2a). To functionalize the nanochannel, two docking sites are extended on the top region for hybridizing the locker (Supplementary Fig. 3), allowing for the creation of nanogatekeeper and membrane-spanning chol-DNGK (Fig. 2b and Supplementary Fig. 4). Atomic force imaging and small-angle X-ray scattering (SAXS) verified this tubular nanostructure (Fig. 2d, Supplementary Table 5, and Supplementary Figs. 6 and 7). PAGE and dynamic light scattering confirmed the successful construction of chol-DNGK, which is in good agreement with the above results (Fig. 2c and Supplementary Fig. 5).

Previous work has shown that the functional groups on both the base and sugar of ATP are involved in a ligand/aptamer interaction^27^. Therefore, to design the locker in this work, we made use of an ATP-binding DNA aptamer sequence to construct the locker, which is also capable of hybridizing the top region of nanogatekeeper (Supplementary Figs. 3 and 8). In the presence of ATP, the higher binding affinity of the locker to ATP results in switching from the closed state nanogatekeeper to the open state. Locker with different concentrations of ATP were studied by circular dichroism spectroscopy to determine differences in attenuation of left and right circularly polarized light passing through a sample (Supplementary Fig. 9). Next, fluorescence spectroscopy was employed to analyze ATP response. In the initial closed state, the fluorescent dye is quenched (top region of nanogatekeeper is labeled with quenchers, while locker is labeled with a fluorophore). Conversely, in the presence of ATP, the locker is released from nanogatekeeper, enabling the “OFF” to “ON” signal readout (Fig. 2e and Supplementary Fig. 10). Different ATP analogs were also tested to study the selectivity of gate-opening (Fig. 2f), revealing good selectivity of ATP response.

### Artificial ion channel mimicry on GPMV

**Fig. 3:**
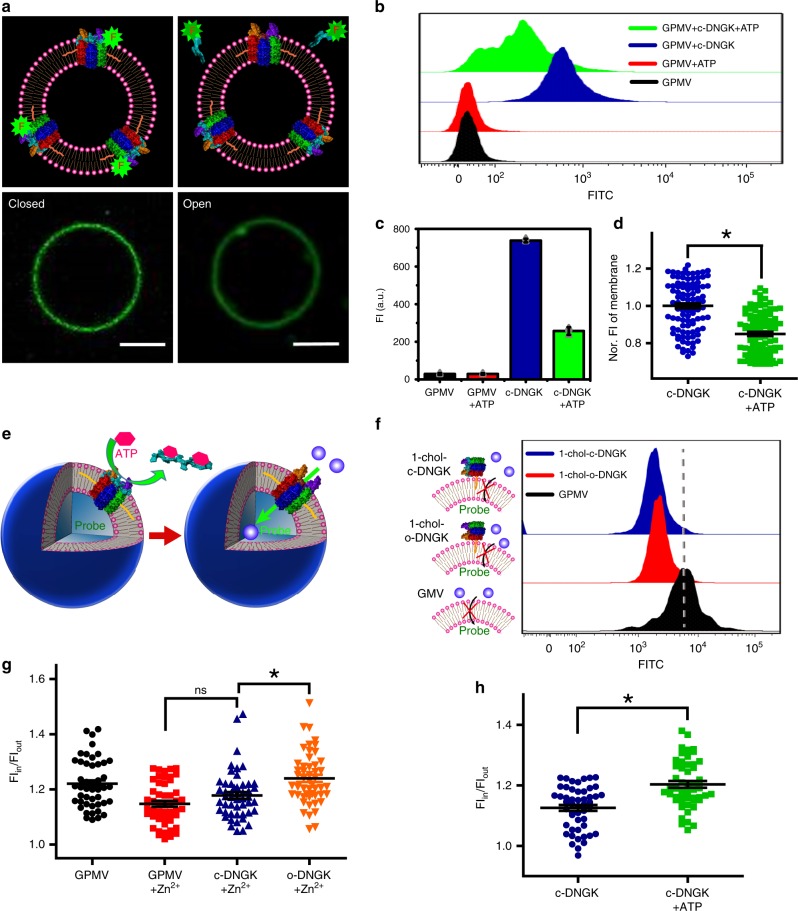
Construction of a membrane-spanning nanogatekeeper on GPMV to mimic cell membrane ion channel. **a** CLSM imaging of localization of Alexa Fluor 488-labeled chol-c-DNGK on the membrane of our giant membrane vesicles. Chol-DNGK anchored on the giant membrane vesicles before (left) or after (right) addition of 3 mM ATP. One micromolar chol-DNGK incubated with 400 μL giant vesicles at 37 °C for 30 min. Scale bar: 5 μm. **b**, **c** Flow cytometry studies of fluorescent intensity change after addition of 3 mM ATP and incubation for 30 min. Decreased fluorescence on the membrane demonstrated that ATP switches the closed state of chol-DNGK to the open state. **d** Statistical evaluation with CLSM. Each column represents the statistical sample population of 100 membrane vesicles corresponding to each group in **a**. **e** Three-dimensional (3D) schematic representation of an ATP-driven chol-DNGK on the membrane of GPMV. **f** Flow cytometry study of one cholesterol-labeled nanogatekeeper (1-chol-c-DNGK and 1-chol-o-DNGK) on GPMV membrane. **g** A 200 μL GPMV solution incubated with 3 μM FluoZin™-3 indicator, followed by adding 0.5 μM chol-DNGK s for 30 min and, finally, treating with 0.2 mM Zn^2+^. Each column represents the statistical sample population of 50 membrane vesicles. **h** Dynamic study of ATP response on chol-c-DNGK directly showing that the closed state switches to the open state after treatment with ATP. A 200 μL solution of GPMVs with 3 μM FluoZin™-3 indicator was incubated for 2 h. Then 0.5 μM nanogatekeeper was incubated for 30 min, followed by the addition of 0.2 mM Zn^2+^ and 3 mM ATP and incubation for 20 min. Each column represents the statistical sample population of 50 membrane vesicles. *P*-values were calculated by Student’s *t*-test. Source data are provided in the Source Data file.

The on-membrane study of chol-DNGK for ion channel mimicry was performed on our previously reported cell-mimicking GPMV^21^. These micro-scaled vesicles are stable and easily engineered by DNA molecules (Supplementary Fig. 11). To demonstrate on-membrane ATP response of chol-DNGK, the feasibility of membrane anchoring of chol-DNGK was initially investigated. Confocal laser scanning microscopy (CLSM) was employed to image membrane anchoring, and the results showed chol-DNGK to be freely moving and homogeneously distributed on the membrane (Supplementary Fig. 12). In this part, the locker is labeled with fluorophore, while nanogatekeeper is free-labeled (Fig. 3a). Driven by ATP, the closed state of chol-DNGK switches to the open state, while by the release of locker, the fluorescence intensity on GPMVs is decreased, transmitting an on-membrane signal from “ON” to “OFF” (Figs. 3b–d).

To study the influence of cholesterol molecule numbers on nanogatekeeper for membrane insertion, we constructed nanogatekeeper labeled with one cholesterol molecule (1-chol-DNGK), but no fluorescence signal increase was observed in either its open state (1-chol-o-DNGK) or its closed state (1-chol-c-DNGK, Fig. 3f), due to the fact that it did not fully span the membrane^22^. So, four cholesterol-labeled nanogatekeeper was used.

After verifying the gate-opening process driven by ATP, we studied the ion channel mimicry of chol-DNGK (Fig. 3e). FluoZin™-3 is a Zn^2+^-selective indicator, and we initially incubated this sensitive commercial organic probe in the chamber of our giant membrane vesicles for zinc ion detection. When the closed state of chol-DNGK switches to the open state, zinc ions diffuse into the core of giant vesicles, turning fluorescence on. We statistically evaluated the fluorescence signal of the encapsulated FluoZin™-3 indicator in two different states of chol-DNGK (Fig. 3g). Upon opening the membrane gate, the fluorescent probe was activated, resulting in the appearance of the inside-to-outside ratio of fluorescence intensity of GPMVs (FI_in_/FI_out_). Importantly, limited ionic leakage confirmed the membrane integrity of such GPMV (Fig. 3g and Supplementary Fig. 15).

Next, we used CLSM to study the gate-opening process in the context of dynamic ATP-triggering via fluorescence detection. In the presence of ATP, chol-c-DNGK switched to the open state and turned on the fluorescence of FluoZin™-3 indicator (Fig. 3h), thus demonstrating that ion channel mimicry had been achieved. This approach was also performed on a giant unilamellar vesicle (GUV), as a common synthetic membrane model (Supplementary Figs. 13 and 14). Although this is used as a potential platform, the lack of inherent complexity found in natural cell membranes requires a more biomimetic platform. Compared to other artificial ion channels^28–31^, this simple and small functional DNA nanostructure is easily designed and economical, and reveals wide mimetic applications.

### Fabrication of artificial molecular signaling networks

**Fig. 4:**
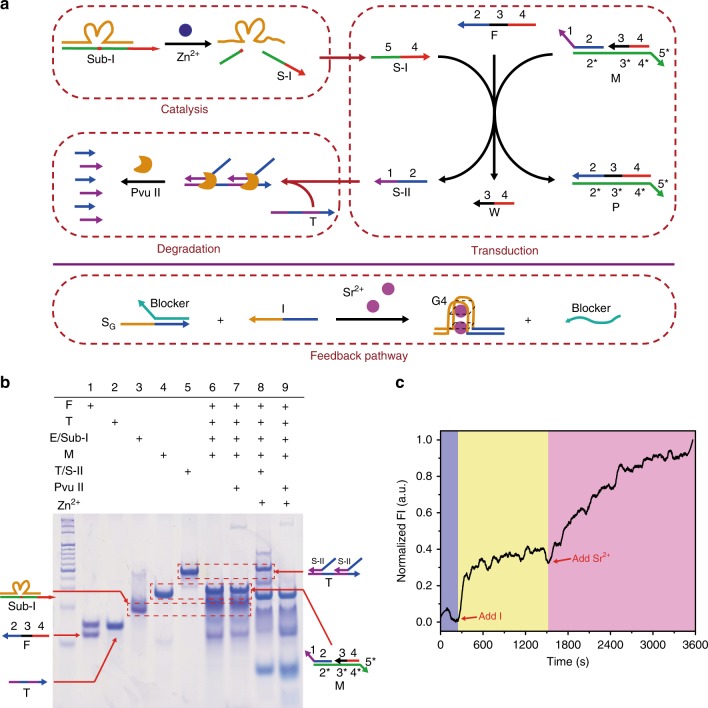
Construction of an artificial molecular signaling network. **a** Schematic representation of signaling network downstream modules and feedback module. Red dashed box indicates each confined reaction module. **b** Twelve percent PAGE analysis of signaling network modules, including ion-mediated catalytic reaction, a DNA circuit reaction and protein enzyme-assisted DNA degradation. DNA cascade reactions were performed in a 37 °C water bath. Lane 1: F strand; Lane 2: T strand; Lane 3: Hybrids of E and Sub-I (E/Sub-I); Lane 4: M strand; Lane 5: Hybrids of T and S-II (T/S-II); Lane 6: Former four DNA reagents; Lane 7: Sample in lane 6 treated with Pvu II; Lane 8: Samples of lane 6 adding zinc ion; Lane 9: Samples of lane 6 treated with Pvu II and zinc ion. Plus (“+”) mark indicates the presence of the corresponding molecule. **c** Fluorescence kinetics showing the dynamic DNA strand displacement of feedback pathway. As shown by the red arrow, the 20 nM strand I and 10 mM Sr^2+^ were successively added into the 20 nM S_G_R duplex solution. Source data are provided in the Source Data file.

As described above, the signaling network involves the integration of two components, including ATP-driven membrane-spanning nanogatekeeper and a confined signaling network that consists of DNA cascades. After confirming the feasibility of the nanogatekeeper, which participates in reception, we next designed the downstream signaling network. To accomplish this, several DNA-based modules were further rationally constructed to carry out the basic roles of cell signaling, transduction and response (Fig. 4a, upper).

We first performed a stepwise characterization of each module. The ion-mediated catalytic reaction was designed on the basis of Zn^2+^-dependent DNAzyme (Supplementary Fig. 16). In the presence of Zn^2+^, DNAzyme (E) cleaved the initial substrate (Sub-I) into two different segments, including the initial signal (S-I) for downstream triggering. PAGE verified the specific cleavage effect (Supplementary Fig. 17), and the titration curve of Zn^2+^-mediated catalytic reaction was revealed by fluorescence spectroscopy (Supplementary Fig. 18). The secondary signaling transduction module was based on the reaction of entropy-driven DNA circuitry that mechanically circulated (Supplementary Figs. 19 and 20). With this module, signal S-I was transmitted to secondary signal (S-II). We analyzed this molecular transduction module by the change in fluorescence intensity (Supplementary Figs. 21–23). The upper right box in Fig. 4a shows that a signal S-II was produced, followed by S-II-triggered protein enzyme-assisted target DNA degradation, indicating the response module. Sensing S-II, the target strand (T) was labeled, forming a DNA hybrid (T/S-II). Thus, this double-stranded hybrid could be specifically recognized by endonuclease Pvu II and degraded into several segments (Supplementary Fig. 24). Two-stage signaling transduction was analyzed together by fluorescence spectroscopy (Supplementary Fig. 26). The successful integration of the above three modules was verified by PAGE (Fig. 4b). Finally, to optically facilitate sensing of the enzymatically degraded segments, a RCA reaction with SYBR Green I (SG-I) was employed to detect the final products from degradation (Supplementary Fig. 25). The RCA probe was created (Supplementary Fig. 27), and the amplified result could be detected by agarose gel (Supplementary Fig. 28). As the output result, an increasing fluorescence signal was observed due to the formation of highly compact and stacked RCA product that exhibits higher sensitivity with SG-1 (Supplementary Fig. 29)^32^.

Meanwhile, we designed a confined feedback reaction in GPMV to regulate the on-membrane nanogatekeeper. Initially, a parallel responsive feedback pathway was investigated (Fig. 4a, bottom). Upon opening of the mimetic ion channel, the influx of strontium ions into the membrane vesicles, together with zinc ions, triggered a toe-hold-mediated DNA strand displacement reaction, thereby releasing a single-stranded DNA, termed blocker, which can plug the bottom region of nanogatekeeper (Fig. 1b and Supplementary Figs. 30–33)^33^. The fluorescence kinetics verified this dynamic process (Fig. 4c). Upon release of the blocker, the bottom region of chol-o-DNGK was plugged by hybridization of the blocker, thereby returning nanogatekeeper to the closed state (Supplementary Fig. 34). Collectively, these results indicated that the feasibility of the confined molecular signaling network had been proven through the integration of modular DNA cascade reactions, as well as the introduction of a feedback pathway.

### An artificial signaling system in biomimetic GPMVs

**Fig. 5:**
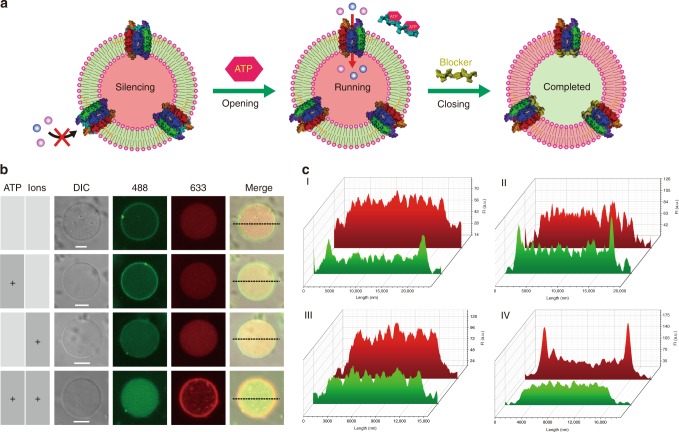
Characterization of artificial molecular signaling system. **a** Schematic illustration of AMSsys working principle. Initially, chol-c-DNGK with biomimetic GPMV effectively shields the extracellular zinc ions. Driven by ATP, however, chol-c-DNGK switches to the open state, allowing ions to freely flow into the chamber by diffusion and trigger the signaling network. Eventually, the feedback pathway mediates chol-o-DNGK to switch back to the original closed state. Simultaneously, reactions of confined signaling network have been accomplished. **b** CLSM analysis of AMSsys in the above different states. Panel 1: without ATP and ions. Confined signaling network stays inactivated: Cy5-labeled (633 channel) blocker is free in the chamber, where the 488 signal saw only minor increase. Panel 2: Addition of ATP drives the opening of chol-c-DNGK, while the signaling network continues to remain inactive by the absence of ion influx triggering. Panel 3: Addition of only ions (no ATP) cannot trigger the confined reactive network and chol-DNGK remains closed. Panel 4: Only in the presence of both ATP and ion influx, can all designed reactions be completed: chol-c-DNGK switches to chol-o-DNGK, followed by diffusion of ions through the mimetic nanochannel, triggering, in turn, the confined signaling network. After completion of the confined reaction cascade, the fluorescent signal from SG-I is revealed (light green spot) and Cy5-labeled blocker plugs the nanogatekeeper (red circle). Plus (“+”) mark denotes that the corresponding trigger (ATP or/and ions) was present. Scale bar, 5 μM. **c** Fluorescence intensity of the white dashed line section of each group in **b**. Groups I, II, III, and IV, respectively, represent the independent groups (from panel 1 to panel 4) in **b** from top to bottom. The green pattern shows the SG-I fluorescence channel, while the red pattern shows the Cy5 fluorescence channel. Source data are provided in the Source Data file.

Encapsulated materials were transported into the vesicle chamber by electroporation (Supplementary Figs. 35–37 and Supplementary Tables 6–8), and DNGK was added; then, a primary system without the feedback pathway was obtained (Supplementary Fig. 38). Comparing with initial high density of vesicles, only a few GPMVs survived after this electroporation. Next, the feedback pathway was integrated into this primary system, and concurrent with all modules, it released a Cy5-labeled blocker against nanogatekeeper (red signal in Fig. 5b).

To regulate the process of cellular response to environmental cues, we tested this integrated AMSsys for stimulation-feedback regulation (Fig. 5a). Driven by ATP, the closed state of chol-DNGK switched to the open state. The influx of ions activated the signaling network, and all confined cascade reactions became operational. Afterwards, chol-DNGK was closed by a released Cy5-labeled blocker. Upon activation, a fluorescence signal on the membrane of GPMV (green ring) originated from the interaction of SG-I molecule with the compact chol-DNGK and the GPMV membrane. After triggering all of the confined reactions, a final product was detected by RCA. The high sensitivity of the compact RCA product combined with SG-1 produced a higher fluorescence intensity, which is shown as a light green spot in Fig. 5b.

As expected, the experimental results confirmed the successful integration of AMSsys. Initially, nanogatekeeper is closed, and the confined signaling network is inactive (Fig. 5b, panel 1). Upon the introduction of ATP, the nanogatekeeper switches to the open state (panel 2). Then the influx of ions triggers the confined reactions, followed by closing of nanogatekeeper by release of blocker from feedback pathway (panel 4). In the absence of ATP, the confined network remains inactive, and ions are shielded by the GPMV membrane (panel 3). Fluorescence intensities of the middle cross section of each vesicle in different states were measured (Fig. 5c and Supplementary Fig. 39), and the results show that the confined molecular signal processing in this artificial system performed efficiently, primarily because of a molecularly crowded environment^34,35^.

## Discussion

Engineering an artificial molecular signaling system involves the design and construction of different functional modules into a rationally integrated system that mimics the cascade of molecular events that finally results in cellular response. To accomplish this, AMSsys was based on a giant membrane vesicle that contains a mimetic ion channel in its membrane with a downstream intravesicular signaling network. This study demonstrates a modular design that utilizes DNA nanotechnology to integrate a membrane-spanning DNA nanostructure and a confined set of DNA cascade reactions allowing crosstalk among modules that mimics the basic elements of cell signaling: reception, transduction and response. In the future, such DNA-based AMSsys may find applications in biosensing, regulation in living systems, and sustainable biomass production and may offer a strategy for engineering an even smarter molecular system and artificial cells.

## Methods

### Construction of DNA nanostructures

All DNA nanostructures were assembled in 1 × TAE/Mg^2+^ buffer (40 mM Tris, 20 mM acetic acid, 1 mM EDTA, pH = 7.4, 12.5 mM Mg^2+^) annealed from 95 to 4 °C with the temperature gradient shown in Supplementary Table 4. The DNA sequence used for DNA nanogatekeeper design is shown in Supplementary Table 2, also see Supplementary Methods.

### PAGE for DNA nanostructure characterization

A 12% native polyacrylamide gel was prepared with 7.4 mL of ultrapure water, 1.5 mL of 10 × TAE/Mg^2+^ (Tris−acetate−EDTA with magnesium ion), 6.0 mL of 30% acryl-bis, 0.11 mL of 10% APS, and 0.010 mL of TEMED. DNA nanostructures were eventually quantified in a volume of 10 μL to give a desired concentration. Then, 2 μL of 6 × loading buffer was directly added to each sample for electrophoresis experiments. Electrophoresis was carried out in fresh 1 × TAE/Mg^2+^ buffer (40 mM Tris-HAc, 1 mM EDTA, and 12.5 mM Mg(Ac)_2_, adjusted to pH 7.4) at 110 V surrounded by an ice-water bath. After stopping electrophoresis, the gel was removed, and DNA bands were stained by Stains-All for 10 min, followed by washing the gel with water. Imaging and analysis were carried out using a Bio-Rad molecular imager with imaging software under ultraviolet light.

### Preparation of cell-mimicking giant membrane vesicle

The cell-mimicking giant membrane vesicles were obtained from detached HeLa or HepG2 cells. Cells were obtained from ATCC (American Type Culture Collection, Manassas, VA, USA) and cultured in RPMI-1640 cell medium (Life Technologies, USA) supplemented with 10% fetal bovine serum (FBS, Gibco) and 1% penicillin (100 U/mL) -streptomycin (100 µg/mL, Life Technologies, USA) in a cell culture incubator at 37 °C with 5% CO_2_ atmosphere. Cell density was determined using a hemocytometer. For adherent HeLa cells, short-term (30 s to 1 min) trypsin treatment was adopted to dissociate cells from the culture flask or dish. Giant membrane vesicles were derived from HeLa cells as previously reported. Briefly, HeLa cells were washed with Dulbeccoʼs phosphate buffered saline (DPBS) four times after 48 h growth and then incubated in phenol red-free RPMI-1640 culture medium containing carboxylfullerenes for 4 h at 37 °C. After removing cell medium, cells were washed with DPBS four times and added to 4 mL of RPMI-1640 culture medium (1×, without phenol red). Then, the adherent cells were irradiated under white light for another 4 h. After overnight incubation, micron-scale giant membrane vesicles were suspended in the supernatant solution, and the collected supernatant fluid was used as prepared.

### AFM imaging

Atomic force microscopy of samples was observed on a Multimode 8 (Bruker, USA) using ScanAsyst mode and imaging in solution. Solution of 5 μL 30 mM Ni^2+^ covered the surface of freshly cleaved mica, and then a 10 µL chol-DNGK sample was added onto the mica surface. After incubation for 5 min at room temperature, the sample was imaged.

### Confocal laser scanning microscopy imaging

We chose CLSM to visualize the behavior of DNA nanostructures on giant membrane vesicles. Typically, a 500 µL solution of membrane vesicles was placed inside a 20 mm confocal dish and incubated with nanogatekeeper at 37 °C for 0.5 h. After incubation, membrane vesicles were directly observed by CLSM imaging.

### Flow cytometry

For ATP response of nanogatekeeper on GPMV membrane, 100 nM nanogatekeeper was incubated with a 400 µL GPMV solution at 37 °C for 30 min. After addition of 3 mM ATP, GPMVs were suspended for flow cytometry analysis on the BD FACSVerse™ flow cytometer by counting 4000 events. To study 1-chol-DNGK, 1.2 µL of 500 µM FluoZin™-3 indicator was incubated with a 200 µL GPMV solution at 37 °C for 2 h. Detection was performed after incubation of 40 µL of 2.5 µM 1-chol-DNGK for 30 min, followed by the addition of 3.2 µL 12.5 mM zinc ion and another incubation for 20 min.

### Statistics

Each measurement was repeated at least three times with triplication for each sample tested. The results are presented as mean ± standard deviation, unless otherwise indicated. Statistical mean differences were evaluated using the unpaired Student’s *t-*test with GraphPad software, and *P* < 0.05 was considered statistically significant.

### Reporting summary

Further information on research design is available in the Nature Research Reporting Summary linked to this article.

## Supplementary information

Reporting Summary

Supplementary Information

## Data Availability

The data that support the findings of this study are available from the corresponding author upon reasonable request. Source data for the figures are provided in the Source Data file.

## Source data

Source Data
