# Supplementary material for: DNA-based artificial molecular signaling system that mimics basic elements of reception and response
Source: Nat Commun. 2020 Feb 20;11:978. doi: 10.1038/s41467-020-14739-6 (PMC7033183; doi:10.1038/s41467-020-14739-6)
Supplement: Supplementary file 1 — Reporting Summary [file 41467_2020_14739_MOESM1_ESM.pdf]

nature research

Corresponding author(s): Weihong Tan

Last updated by author(s): Dec 15, 2019

Reporting Summary

Nature Research wishes to improve the reproducibility of the work that we publish. This form provides structure for consistency and transparency in reporting. For further information on Nature Research policies, see Authors & Referees and the Editorial Policy Checklist .

Statistics

For all statistical analyses, confirm that the following items are present in the figure legend, table legend, main text, or Methods section.

n/a	Confirmed	
☐	☒	The exact sample size (*n*) for each experimental group/condition, given as a discrete number and unit of measurement
☐	☒	A statement on whether measurements were taken from distinct samples or whether the same sample was measured repeatedly
☐	☒	The statistical test(s) used AND whether they are one- or two-sided<br>*Only common tests should be described solely by name; describe more complex techniques in the Methods section.*
☐	☒	A description of all covariates tested
☐	☒	A description of any assumptions or corrections, such as tests of normality and adjustment for multiple comparisons
☐	☒	A full description of the statistical parameters including central tendency (e.g. means) or other basic estimates (e.g. regression coefficient) AND variation (e.g. standard deviation) or associated estimates of uncertainty (e.g. confidence intervals)
☐	☒	For null hypothesis testing, the test statistic (e.g. *F*, *t*, *r*) with confidence intervals, effect sizes, degrees of freedom and *P* value noted<br>*Give P values as exact values whenever suitable.*
☒	☐	For Bayesian analysis, information on the choice of priors and Markov chain Monte Carlo settings
☒	☐	For hierarchical and complex designs, identification of the appropriate level for tests and full reporting of outcomes
☒	☐	Estimates of effect sizes (e.g. Cohen's *d*, Pearson's *r*), indicating how they were calculated

Our web collection on statistics for biologists contains articles on many of the points above.

Software and code

Policy information about availability of computer code

Data collection	Fluorescence spectral data was measured on Fluoro Max-4 (Horiba Jobin Yvon). PAGE gel imaging data was collected using Microtek's Bio-6000 scanner and Bio-Rad ChemiDoc XRS System. Flow cytometry data was collected on the BD FACSVerse™ flow cytometer. Confocal imaging data was collected on Zeiss LSM 880 and Olympus FV1000 confocal laser scanning microscope.
Data analysis	Fluorescence spectral data was analyzed with OriginLab 2016. Statistical mean differences were evaluated using the unpaired Student's t-test with GraphPad 5. Flow cytometry data was analyzed using FlowJo 7.6.2. Confocal imaging data was analyzed using live softwares on Zeiss LSM 880 and Olympus FV1000 confocal laser scanning microscope.

For manuscripts utilizing custom algorithms or software that are central to the research but not yet described in published literature, software must be made available to editors/reviewers. We strongly encourage code deposition in a community repository (e.g. GitHub). See the Nature Research guidelines for submitting code & software for further information.

Data

Policy information about availability of data

All manuscripts must include a data availability statement. This statement should provide the following information, where applicable:

- Accession codes, unique identifiers, or web links for publicly available datasets
- A list of figures that have associated raw data
- A description of any restrictions on data availability

The data that support the plots within this paper and other findings of this study are available from the corresponding author upon reasonable request.

Field-specific reporting

Please select the one below that is the best fit for your research. If you are not sure, read the appropriate sections before making your selection.

- [x] Life sciences
- [ ] Behavioural & social sciences
- [ ] Ecological, evolutionary & environmental sciences

For a reference copy of the document with all sections, see nature.com/documents/nr-reporting-summary-flat.pdf

Life sciences study design

All studies must disclose on these points even when the disclosure is negative.

Sample size	Due to the high reproducibility and consistency between generating cell-mimicking giant vesicles from living cells, it was predetermined that a sample size of at least n=3 would allow for adequate analysis to reach meaningful conclusions of the data.
Data exclusions	No data was excluded from studies.
Replication	All replication of experiments was successful; however, in the case of replication studies of AMSsys construction (Fig. 5), replication was not fully possible due to limited vesicles were survived after electroporation.
Randomization	Throughout the whole experiment, samples were randomized into groups.
Blinding	No blinding was used throughout experiments. All data collected was quantifiable and blinding would not change any bias in data collected.

Reporting for specific materials, systems and methods

We require information from authors about some types of materials, experimental systems and methods used in many studies. Here, indicate whether each material, system or method listed is relevant to your study. If you are not sure if a list item applies to your research, read the appropriate section before selecting a response.

Materials & experimental systems

n/a	Involved in the study	
x		Antibodies
	x	Eukaryotic cell lines
x		Palaeontology
x		Animals and other organisms
x		Human research participants
x		Clinical data

Methods

n/a	Involved in the study	
x		ChIP-seq
	x	Flow cytometry
x		MRI-based neuroimaging

Eukaryotic cell lines

Policy information about cell lines

Cell line source(s)	HeLa and HepG2 cells were obtained from ATCC.
Authentication	Cell cultures purchased from ATCC were authenticated by Short Tandem Repeat (STR) prior to purchase.
Mycoplasma contamination	Cell lines were not tested for mycoplasma contamination.
Commonly misidentified lines (See ICLAC register)	No misidentified line for neither Hela cell nor HepG2 cell. Contamination of cells will not affect the generation of cell-mimicking giant vesicles.

Flow Cytometry

Plots

Confirm that:

- [ ] The axis labels state the marker and fluorochrome used (e.g. CD4-FITC).
- [x] The axis scales are clearly visible. Include numbers along axes only for bottom left plot of group (a 'group' is an analysis of identical markers).
- [ ] All plots are contour plots with outliers or pseudocolor plots.
- [x] A numerical value for number of cells or percentage (with statistics) is provided.

Methodology

Sample preparation	For ATP response of nanogatekeeper on GPMV membrane, 100 nM nanogatekeeper was incubated with a 400 µL GPMV solution at 37 °C for 30 min. After addition of 3 mM ATP, GPMVs were suspended for flow cytometry analysis on the BD FACSVerse™ flow cytometer by counting 4,000 events. To study 1-chol-DNGK, 1.2 µL of 500 µM FluoZin™-3 indicator was incubated with a 200 µL GPMV solution at 37 °C for 2 h. Detection was performed after incubation of 40 µL of 2.5 µM 1-chol-DNGK for 30 min, followed by the addition of 3.2 µL 12.5 mM zinc ion and another incubation for 20 min.
Instrument	Samples were analyzed by BD FACSVerse™ flow cytometer.
Software	All data was analyzed using FlowJo 7.6.2 with overlay histogram plots produced within FlowJo.
Cell population abundance	During sample measurements, initial gate was used to ensure a cell-mimicking vesicle count of 4,000 events. For GUV, the population is 100,000 events.
Gating strategy	Initial sample populations were gated for a live population using FSC and SSC plot of vesicles only sample. This live population was then used in fluorescent histograms. No gating was applied to histograms.

☒ Tick this box to confirm that a figure exemplifying the gating strategy is provided in the Supplementary Information.
