# Supplementary material for: DNA-based artificial molecular signaling system that mimics basic elements of reception and response
Source: Nat Commun. 2020 Feb 20;11:978. doi: 10.1038/s41467-020-14739-6 (PMC7033183; doi:10.1038/s41467-020-14739-6)
Supplement: Supplementary file 2 — Supplementary Information [file 41467_2020_14739_MOESM2_ESM.pdf]

Supplementary Information

DNA-based Artificial Molecular Signaling System that Mimics Basic Elements of Reception, Transduction, and Response

Ruizi Peng[1,2], Liujun Xu[1], Huijing Wang[1], Yifan Lyu[2,3], Dan Wang[1], Cheng Bi[1], Cheng Cui[1,3,4], Chunhai Fan[2], Qiaoling Liu[1*], Xiaobing Zhang[1*], Weihong Tan[1,3,4*]

[1] Molecular Science and Biomedicine Laboratory (MBL), State Key Laboratory of Chemo/Bio-Sensing and Chemometrics, College of Chemistry and Chemical Engineering, College of Biology, and Aptamer Engineering Center of Hunan Province, Hunan University, Changsha, Hunan 410082, P. R. China.

[2]Institute of Molecular Medicine (IMM), State Key Laboratory of Oncogenes and Related Genes Renji Hospital, Shanghai Jiao Tong University School of Medicine, and College of Chemistry and Chemical Engineering, Shanghai Jiao Tong University, Shanghai 200240, China

[3]Institute of Cancer and Basic Medicine (IBMC), Chinese Academy of Sciences, The Cancer Hospital of the University of Chinese Academy of Sciences, Hangzhou, Zhejiang 310022, China

[4]Foundation for Applied Molecular Evolution, 13709 Progress Boulevard, Alachua, FL 32615, USA.

Corresponding Authors

*E-mail: tan@hnu.edu.cn

xbzhang@hnu.edu.cn

qlliu@iccas.ac.cn

Table of contents

1. Supplementary methods 5

1a. General reagents.. 5

1b. DNA synthesis and purification 7

Supplementary Notes

2. Construction of DNA nanostructures 10

2a. DNA nanochannel design... 10

2b. Theoretical estimation of DNA nanochannel 12

2c. Cholesterol-free nanogatekeeper design 13

2d. Cholesterol-modified nanogatekeeper design 14

2e. DNA nanostructure assembly.. 15

2f. DNA nanostructure characterization 16

3. ATP-responsive nanogatekeeper in solution... 19

3a. Lock strand characterization 19

3b. DNA nanogatekeeper in response to addition of ATP................................... 21

4. Microcompartment preparation 22

4a. Preparation of cell-mimicking giant membrane vesicles 22

4b. Preparation of giant unilamellar vesicle 24

5. Study of nanogatekeeper on cell-mimicking membrane vesicles......................... 25

5a. Dynamic behavior of nanogatekeeper on cell-mimicking membrane vesicles.......... 25

5b. Studies of nanogatekeeper on giant unilamellar vesicles.............................. 26

5c. Ca^{2+} channel mimicry of nanogatekeeper 28

6. In solution construction of DNA networks... 29

6a. Module of ion-mediated catalysis 31

6b. DNA circuit signaling transduction 34

6c. Endonuclease-recognized target degradation... 38

6d. Characterization of integrated modules 39

6e. Rolling circle amplification (RCA) to detect degradation products................. 41

6f. Feedback pathway 44

7. Transport of biomolecules into cell-mimicking vesicles..................................... 48

7a. Purification of fluorescent dye-modified restriction endonuclease............................ 48

7b. Transport of reagents with incubation... 49

7c. Transport of reagents with electroporation 51

8. Regulation of AMSsys without feedback reaction 52

9. Engineering AMSsys in biomimetic GPMVs... 53

Supplementary References 55

Supplementary Table 1. Abbreviations in this study.

Abbreviation	Full name
GPMV	Giant plasma membrane vesicle
GUV	Giant unilamellar vesicle
EDTA	Ethylenediaminetetraacetic acid
DMSO	Dimethyl sulfoxide
SG-I	SYBR Green I
ATP	Adenosine-5'-triphosphate
CTP	Cytidine-5'-triphosphate
GTP	Guanosine-5'-triphosphate
UTP	Uridine-5'-triphosphate
BHQ-1	Black hole quencher-1
PAGE	Polyacrylamide gel electrophoresis
APS	Ammonium persulfate
AFM	Atomic force microscopy
SAXS	Small-angle X-ray scattering
RCA	Rolling circle amplification

1. Supplementary methods

1a. General reagents

Buffer solutions used in this study

All HPLC-purified oligonucleotides and various DNA nanostructure samples were assembled in 1×Tris-acetic acid-EDTA/Mg^{2+} buffer (1×TAE/Mg^{2+}, 40 mM Tris, 20 mM acetic acid, 1 mM EDTA, 12.5 mM Mg^{2+}, pH=7.4) for test tube experiments, but assembled in 10×TAE/Mg^{2+} buffer (pH=8.0) for on-vesicule experiments. 50×TAE/Mg^{2+} buffer (pH=8.4) and 50 mM HEPES (1.5 M NaCl, pH=7.0) were prepared.

Gene Pulser electroporation buffer was purchased from Bio-Rad.

Protein enzymes used in this study

10 U/μL Pvu II (ER0635, Thermo Fisher Scientific), T4 DNA ligase or *E. coli* DNA Ligase (Takara Bio, Dalian), 20 U/μL Exonuclease I (Sangon Biotech, Shanghai), 200 U/μL Exonuclease III (Thermo Fisher Scientific), phi 29 DNA polymerase (Sangon Biotech).

Commercial fluorescent probes used in this study

FluoZin™-3, AM, indicator (Invitrogen) solid was dissolved in DMSO to a final concentration of 1 mM, stored at -20 °C, and protected from light.

SYBR Green I (SG-I) was purchased from Beijing Dingguo Changsheng Biotechnology Co. Ltd. (10,000×) and diluted to 50× for use.

Ethidium bromide was purchased from Thermo Fisher Scientific.

Calcein-AM (Invitrogen) solid was dissolved in 1:1 DMSO: Methanol to a final concentration of 1 mM.

FM™ 4-64 Dye (N-(3-Triethylammoniumpropyl)-4-(6-(4-(Diethylamino) Phenyl) Hexatrienyl) Pyridinium Dibromide) was purchased from Life Technologies (Thermo Fisher Scientific). This lipophilic dye was reported to stain lipid membranes with red fluorescence (excitation/emission maxima ~515/640 nm).

The solutions of ATP, CTP, GTP and UTP were purchased from ShangHai Yuanye Biotechnology

Co., Ltd.

Stains-All was purchased from Sigma-Aldrich Co., Ltd. DNA ladders and 6×DNA loading buffer were purchased from TaKaRa Biotechnology Co., Ltd. (Dalian, China).

Ultrapure water (electric resistance, 18.2 MΩ cm) obtained through a Millipore Milli-Q water purification system (Billerica, MA, USA) was used throughout the experiments.

1b. DNA synthesis and purification

DNA sequences were synthesized on a DNA synthesizer (PolyGen GmbH, Langen, Germany). The synthesis protocol was set up according to the requirements specified by the reagents' manufacturers (12 columns). After on-machine synthesis, the DNA products were deprotected and cleaved from CPG at 65 °C in a water bath and incubated with 2 mL of AMA (ammonium hydroxide and 40% methylamine, 1:1) for normal deprotection for 30min. However, for Cy3- and Cy5-modified DNA strands, 2 mL of mixed solution (methanol: tert-butylamine: water in 1:1:2 ratio) was used for incubation for 3-4 hours after cleavage from CPG. The cleaved DNA product was transferred into a 15 mL centrifuge tube and mixed with 200 µL of 3 M NaCl and 5 mL of ethanol, after which the sample was placed in a freezer at −20 °C for ethanol precipitation. Next, the DNA product was spun at 4,000 rpm at 4 °C for 30 min. The supernatant was removed, and the precipitated DNA product was dissolved in 400 µL of 0.1 M triethylamine acetate (TEAA) for HPLC purification.

HPLC purification was performed with a cleaned C18 column (Inertsil ODS-3, 5 µm, 4.6×250mm, GL Science Inc., Japan) and Agilent 1260 Infinity Quaternary LC (Agilent Technologies, Germany). The collected DNA product was dried and processed for detritylation by dissolving and incubating in 200 µL of 80% acetic acid for 20 min. The detritylated DNA product was mixed with 20 µL of 3 M NaCl and 500 µL of ethanol and placed into a freezer at −20 °C for 30 min. After that, the DNA product was spun at 14,000 rpm at 4 °C for 5 min. The DNA product was dried by a vacuum dryer and dissolved in ultrapure water, followed by desalting with desalting columns.

Among the DNA strands, the phosphothioate group-inserted, cholesterol-labeled and Alexa 488-labeled DNA strands were synthesized and purified by Sangon Biotech (Shanghai) Co., Ltd., and DNAzyme strands were purchased from TaKaRa Biotechnology Co., Ltd. (Dalian, China). All DNA products were quantified and stored in ultrapure water (Milli-Q) for subsequent experiments. The detailed sequences are given in Supplementary Table 2 and 6.

Supplementary Table 2. DNA sequences of DNA nanogatekeeper.

Name	DNA Sequences (5'-3')
1	AGCGAACGTGGAttttGTCCGACATCGGCAAGCTCCCttttTCGACTATT
2-short	CCGATGTCGGACttttACACGATCTTCGCCTGCTGGGttttGGGAGCTTG
3	CGAAGATCGTGTttttCCACAGTTGATTGCCCTTCACttttCCCAGCAGG
4	AATCAACTGTGGttttTCTCACTGGTGATTAGAATGCttttGTGAAGGGC
5-short	TCACCAGTGAGAttttTGTCGTACCAGGTGCATGGATttttGCATTCTAA
6	CCTGGTACGACAttttTCCACGTTCGCTAATAGTCGAttttATCCATGCA
1-chol	AGCGAACGTGGAttttGTCCGACATCGGCAAGCTCCCttttTCGACTATT-cholesterol
3-chol	CGAAGATCGTGTttttCCACAGTTGATTGCCCTTCACttttCCCAGCAGG-cholesterol
4-chol	AATCAACTGTGGttttTCTCACTGGTGATTAGAATGCttttGTGAAGGGC-cholesterol
6-chol	CCTGGTACGACAttttTCCACGTTCGCTAATAGTCGAttttATCCATGCA-cholesterol
2-ATP	CCGATGTCGGACtt**CCCAGGT**tttt**CCTCCGC**ttACACGATCTTCGCCTGCTGGGttttGGGAGCTTG
2Q-ATP	CCGATGTCGGACtt**CCCAGGTT**^{BHQ1}ttt**CCTCCGC**ttACACGATCTTCGCCTGCTGGGTTTTGGGAGCTTG
2-B1	CCGATGTCGGACTTTTACACGATCTTCGCCTGCTGGGTT**GACACTA**TTTT**GATTAGT**TTGGGAGCTTG
2-B2	CCGATGTCGGACTTTTACACGATCTTCGCCTGCTGGGTT**CCTACAGTTTTCGGGCCT**TTGGGAGCTTG
2-ATP-B1	CCGATGTCGGACTT**CCCAGGT**TTTT**CCTCCGC**TTACACGATCTTCGCCTGCTGGGTT**GACACTA**T*T*T*T**GATTAGT**TTGGGAGCTTG
2-ATP-B2	CCGATGTCGGACTT**CCCAGGT**TTTT**CCTCCGC**TTACACGATCTTCGCCTGCTGGGTT**CCTACAG**T*T*T*T**CGGGCCT**TTGGGAGCTTG
5-ATP	TCACCAGTGAGATT**CCCAGGT**TTTT**CCTCCGC**TTTGTCGTACCAGGTG

	CATGGATTTTTGCATTCTAA
5Q-ATP	TCACCAGTGAGATT**CCCAGGT**T^{BHQ1}TTT**CCTCCGC**TTTGTCGTACCAGGTGCATGGATTTTTGCATTCTAA
5-B1	TCACCAGTGAGATTTTTGTCGTACCAGGTGCATGGATTT**CTAGCTG**TTTT**TGAGGAT**TTGCATTCTAA
5-B2	TCACCAGTGAGATTTTTGTCGTACCAGGTGCATGGATTTCCTACAGTTTTCGGGCCTTTGCATTCTAA
5-ATP-B1	TCACCAGTGAGATT**CCCAGGT**TTTT**CCTCCGC**TTTGTCGTACCAGGTGCATGGATTT**CTAGCTG**T*T*T*T**TGAGGAT**TTGCATTCTAA
5-ATP-B2	TCACCAGTGAGATT**CCCAGGT**TTTT**CCTCCGC**TTTGTCGTACCAGGTGCATGGATTT**CCTACAGTTTTCGGGCCT**TTGCATTCTAA
lock	***ACCTGGG***GGAGTATT***GCGGAGG***AAGGTTTT***ACCTGGG***GGAGTATT***GCGGAGG***AAGGT
FAM-lock	FAM-***ACCTGGG***GGAGTATT***GCGGAGG***AAGGTTTT***ACCTGGG***GGAGTATT***GCGGAGG***AAGGT
AF488-lock	Alexa Fluor 488-***ACCTGGG***GGAGTATT***GCGGAGG***AAGGTTTT***ACCTGGG***GGAGTATT***GCGGAGG***AAGGT
SL	FAM-***ACCTGGG***NNNNNNNN***GCGGAGG***NNNNNNNN***ACCTGGG***NNNNNNNN***GCGGAGG***NNNNN

Underlined letters denote the lock sequence binding domain.

Wave underlined letters denote the blocker strand binding domain.

Italic bold letters of lock strands denote binding bases.

Red letters represent the modification including cholesterol, Alexa Fluor 488 and BHQ-1 (black hole quencher-1).

* indicates a phosphothioate group between two thymine nucleotides making up a hydrophobic end of the DNA nanostructure.

N in SL denote the random base.

2. Construction of DNA nanostructures

2a. DNA nanochannel design

Design of the DNA nanogatekeeper was based on a 6-helix barrel-like DNA nanochannel (Supplementary Figure 1). The height of the DNA nanochannel is 21 base pairs having 2 helical turns (10.5 base pairs per turn). Every DNA double strand is separated by four thymines (T) to improve flexibility.

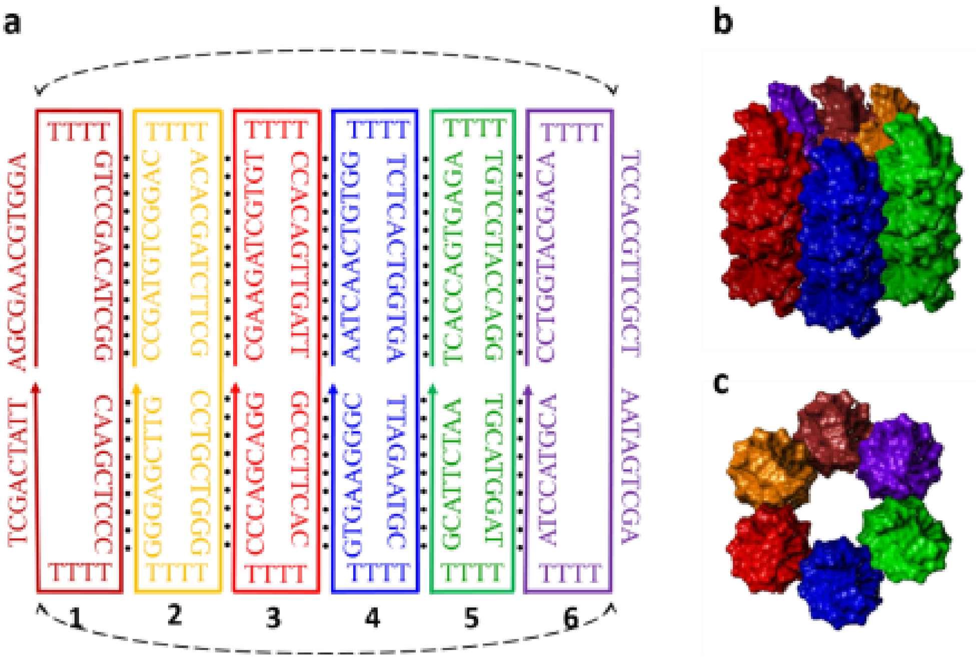

Supplementary Figure 1. The fundamental structure of DNA nanochannel for fabricating DNA nanogatekeeper. (a) 2D map of barrel-like DNA nanochannel. (b) Front view of 3D model of DNA nanochannel. (c) Top view of 3D model of DNA nanochannel.

Supplementary Table 3. Theoretical thermal stability of lateral edges of nanogatekeeper.

Binding domains	ΔG(kcal/mol)	ΔH	ΔS(cal/mol/K)	T_m (°C)
0 (dsDNA 1/2)	-27.4	-169.9	-459.4	73.9
1 (dsDNA 2/3)	-27.0	-167.8	-454.1	73.3
2 (dsDNA 3/4)	-24.2	-164.2	-451.4	67.8
3 (dsDNA 4/5)	-22.2	-159.9	-444.1	64.0
4 (dsDNA 5/6)	-24.9	-166.2	-455.6	69.0
5 (dsDNA 6/1)	-24.4	-164.7	-452.4	68.1
Locker/top dock	-24.0	-199.7	-566.6	61.5°C
Blocker/bottom dock	-15.4	-157.9	-459.5	49.3°C

Table demonstrates that this small DNA nanostructure can be theoretically self-assembled in the given condition. All values were calculated using Mfold software. Strand concentration=1 μM, Mg^{2+}=12.5 mM, Na^{+}=10 mM, Temperature=37 °C. T_m denotes the DNA melting temperature.

2b. Theoretical estimation of DNA nanochannel

Theoretically, the lumen diameter of a 6-helix DNA nanochannel is 2 nm (orange circle), and the calculation is shown in Supplementary Figure 2.

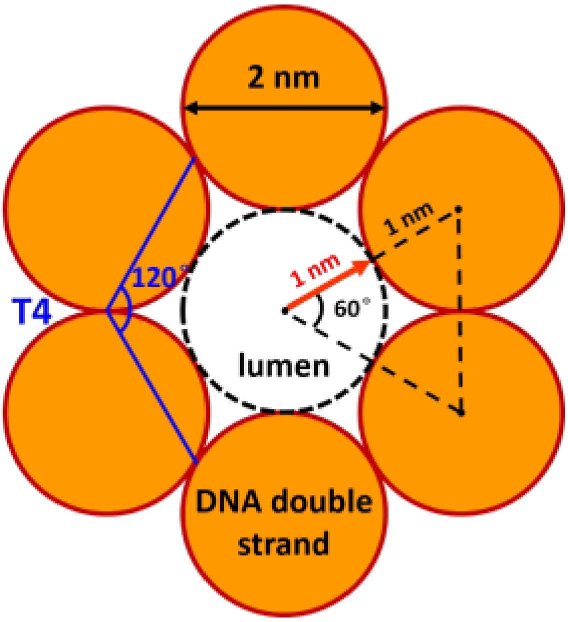

Supplementary Figure 2. Hexagonal geometry of DNA nanochannel. The theoretical estimation for the lumen radius of the DNA nanochannel. The orange circle represents the double-stranded DNA, while the theoretical edge of lumen is circled with a dashed line. In the free state of the buffer solution, the theoretical radium of this nanostructure is 1 nm. Blue letter T4 denotes four thymine nucleotides.

2c. Cholesterol-free nanogatekeeper design

The cholesterol-free DNA nanogatekeeper was designed based on a 6-helix bundle DNA nanochannel. Two different docking sites were extended in strands 2 and 5 as shown in Supplementary Figure 2, respectively, termed strand 2-ATP and 5-ATP.

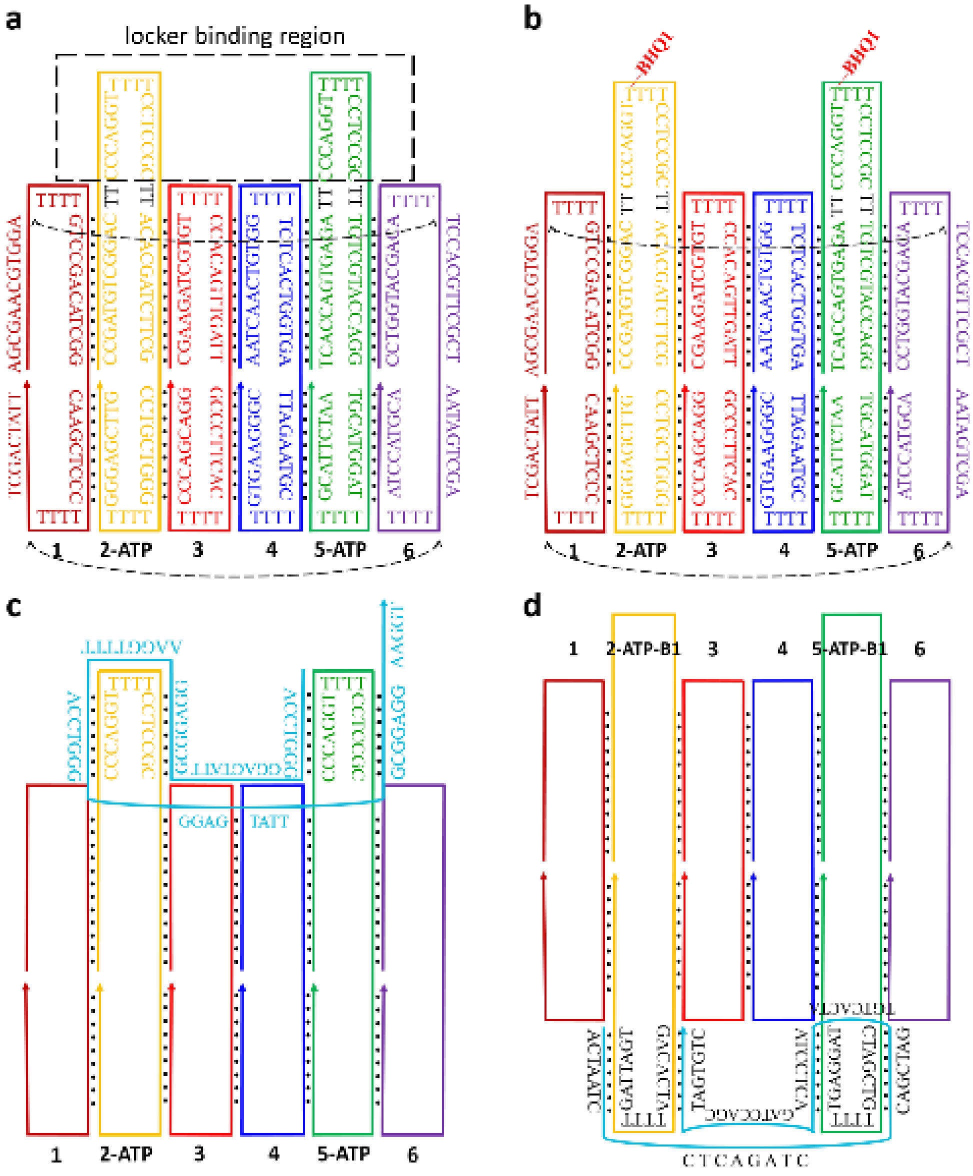

Supplementary Figure 3. DNA nanogatekeeper was fabricated based on DNA nanochannel. The locker binding region (black dashed box) was designed. (a) 2D sequence map of DNA nanogatekeeper. (b) Position of the quencher BHQ-1 (Int BHQ-1 dT) on previous nanostructure (panel a). 2D sequence map shows the binding domain of locker (c) and blocker (d) on DNA nanogatekeeper.

2d. Cholesterol-modified nanogatekeeper design

To create a membrane-spanning DNA nanostructure, we assembled a cholesterol-modified DNA nanogatekeeper in a test tube by programmable annealing. Four cholesterol molecules were respectively modified in the 3'-end of strand 1, strand 3, strand 4 and strand 6 (Supplementary Table 2). These modified positions are in the middle of the nanostructure.

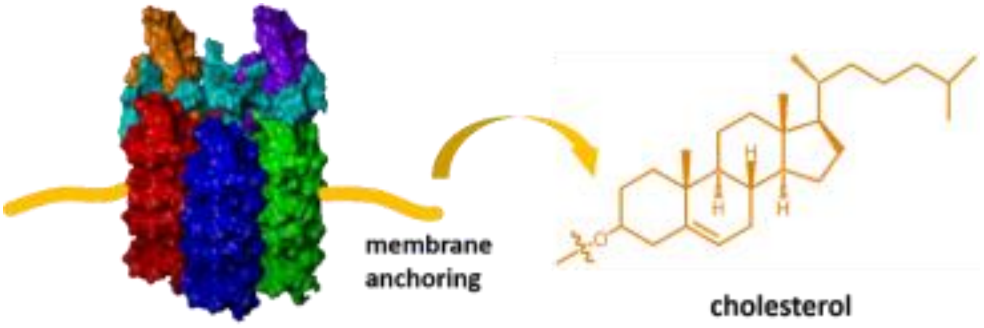

Supplementary Figure 4. Schematic representation of cholesterol molecule (orange)-modified nanogatekeeper (close state). This amphipathic DNA nanostructure spans the membrane by hydrophobic insertion of cholesterol.

2e. DNA nanostructure assembly

All DNA nanostructures were assembled in 1×TAE/Mg^{2+} (40 mM Tris, 20 mM acetic acid, 1 mM EDTA, 12.5 mM Mg^{2+}, pH=7.4) buffer annealed from 95 ºC to 4 ºC with the temperature gradient shown in Supplementary Table 4. The DNA sequence of DNA nanogatekeeper design is shown in Supplementary Table 2.

Supplementary Table 4. Detailed annealing program for nanogatekeeper assembly.

Steps	Temperature range	Gradient	Cycling Times
1	95 °C	2 min	Hold
2	95-85 °C	-1 °C /min	10
3	85-35 °C	-0.1 °C / min	500
4	35-4 °C	-1 °C /min	31
5	4 °C	forever	hold

2f. DNA nanostructure characterization

Native polyacrylamide gel electrophoresis (PAGE) for DNA nanostructure characterization

A 12% native polyacrylamide gel was prepared with 7.4 mL of ultrapure water (Milli-Q), 1.5 mL of 10×TAE/Mg^{2+}, 6.0 mL of 30% acryl-bis, 0.11 mL of 10% APS (fresh), and 0.010 mL of TEMED. DNA nanostructures were eventually quantified in a volume of 10 μL to give a desired concentration. Then, 2 μL of 6× loading buffer was directly added to each sample for electrophoresis experiments. Electrophoresis was carried out in fresh 1×Tris−acetate−EDTA (1×TAE/Mg^{2+}) buffer (40 mM Tris-HAc, 1 mM EDTA, and 12.5 mM $Mg(Ac)_2$, adjusted to pH 7.4) at 110 V surrounded by an ice-water bath. After stopping electrophoresis, the gel was removed, and DNA bands were stained with Stains-All for 10 min, followed by washing the gel with water. Imaging and analysis were carried out using a Bio-Rad molecular imager with imaging software under UV light.

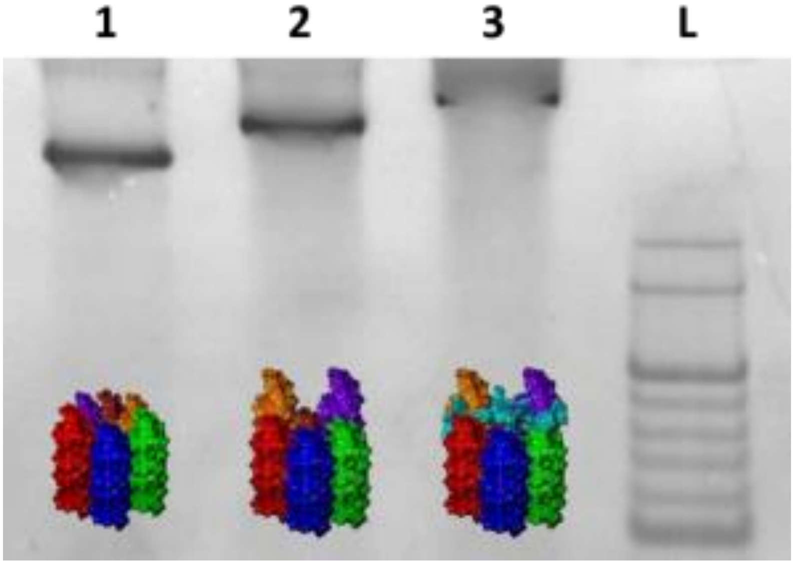

Supplementary Figure 5. PAGE (12%) analysis of assembly of cholesterol-free nanogatekeeper in buffer, running 110 V for 2 h in an ice-water bath. All sample volume is 10 μL with concentration of 2 μM. Lane 1: DNA nanochannel, Lane 2: open state nanogatekeeper (o-DNGK), Lane 3: closed state nanogatekeeper (c-DNGK). L: 20 bp ladder consisting of double strands of DNA with length increasing in 20 bp steps. All DNA bands were stained with Stains-All and then imaged using the Bio-Rad ChemiDoc XRS System.

AFM imaging.

Atomic force microscopy of samples was observed on a Multimode 8 (Bruker, USA) using ScanAsyst mode and imaging in solution. Solution of 5 μL 30 mM Ni^{2+} covered the surface of freshly cleaved mica, and then a 10 µL chol-DNGK sample was added onto the mica surface. After incubation for 5 min at room temperature, the sample was imaged.

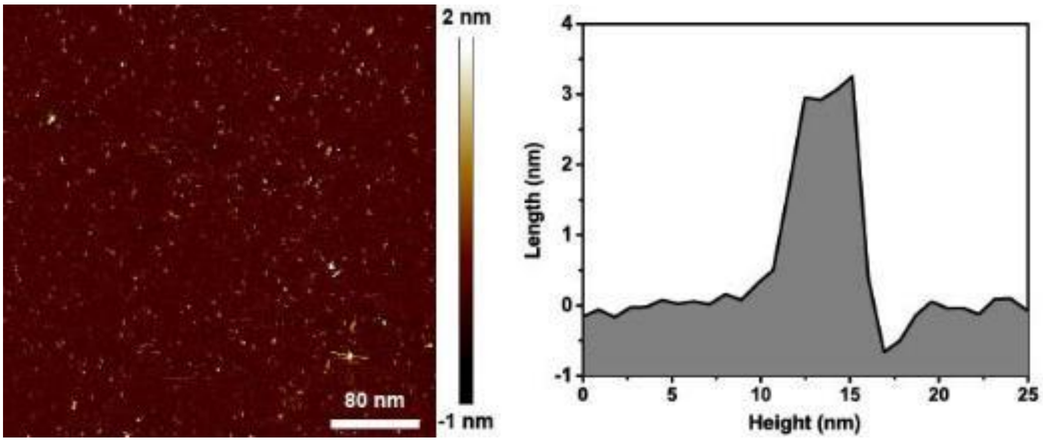

Supplementary Figure 6. (a) AFM image of cholesterol-modified nanogatekeeper on mica. (b) Profile of one chol-DNGK in cross-section.

Small-angle X-ray scattering (SAXS)

SAXS has been proven a powerful technique to detect conformational change and ligand binding of DNA nanostructures.

All nanogatekeeper samples for SAXS experiments were purified by 12% PAGE. Then, dynamic light scattering (DLS) was employed for verification of their monodispersity. SAXS measurements were performed on beamline BL19U2 of the National Center for Protein Science Shanghai (NCPSS) at the Shanghai Synchrotron Radiation Facility (SSRF). The distance from detector to sample is 2644 mm. The maximum value of momentum transfer q reaches to 0.35 $Å^{-1}$ ($q = 4\pi\sin\theta/\lambda$, where 2θ is the scattering angle). The measurement was performed at room temperature, 25 °C.

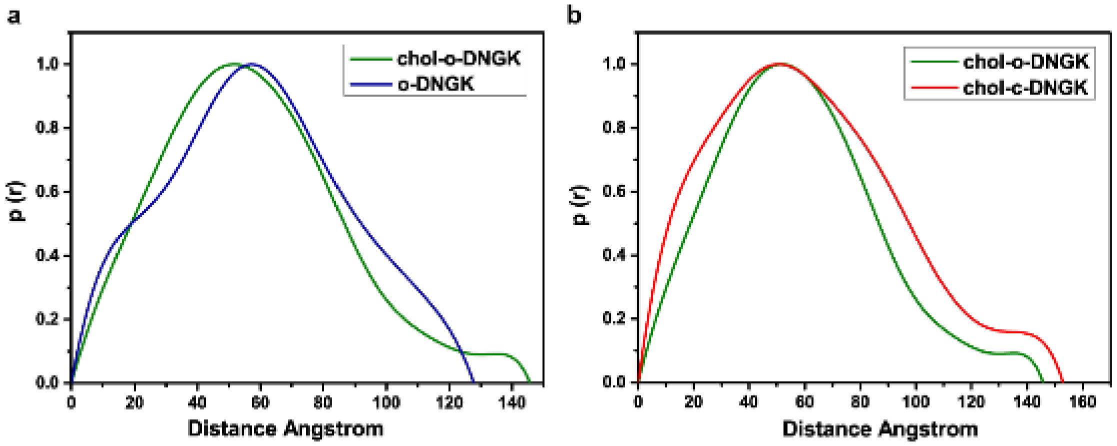

Supplementary Figure 7. SAXS clearly revealed the tubular feature of nanogatekeeper. (a) Cholesterol modification significantly changed the overall shape of nanogatekeeper. The weak peak between 100 to 140 angstrom was caused by cholesterol molecules that attached onto this tubular nanostructure. (b) The shoulder peak on the left indicates the hybridization of locker strands onto the nanogatekeeper nanostructure.

P (r): Pair distance distribution function.

Supplementary Table 5. SAXS study of nanogatekeeper

Nanostructures	R_g (Å)	D_{max} (Å)
o-DNGK	41	133
chol-o-DNGK	46	146
chol-c-DNGK	50	158

R_g and D_{max} values clearly revealed that the cholesterol-modified nanogatekeeper (chol-DNGK) at its open state has larger overall volume than that of unmodified nanogatekeeper. This agreed well with the hydrodynamic size measured from DLS. Hybridization of locker (57 nt) changed the geometric morphology of nanogatekeeper, as presented in the scattering curves of nanogatekeeper in its open and closed states. Meanwhile, hybridization of locker further increased the total volume of nanogatekeeper, demonstrating the formation of c-DNGK based on o-DNGK.

R_g: Radius of gyration; D_{max}: The maximum distance between two points within an object.

3. ATP-responsive nanogatekeeper in solution

3a. Lock strand characterization

Lock strand design. The design of lock strand was based on ATP aptamer. Sequence of original ATP binding aptamer:

5'-ACC TGG GGG AGT ATT GCG GAG GAA GGT-3'.

The lock strand possesses two repeats of the above ATP sequences separated by three thymine nucleotides (Supplementary Table 2). Interestingly, the sequences of the top region of nanogatekeeper (extension part in strand 2-ATP and 5-ATP) are the same as those of the lock strand containing two repeats. Thus, the locker can hybridize the top region of nanogatekeeper in a symmetrical manner (Supplementary Fig. 8b).

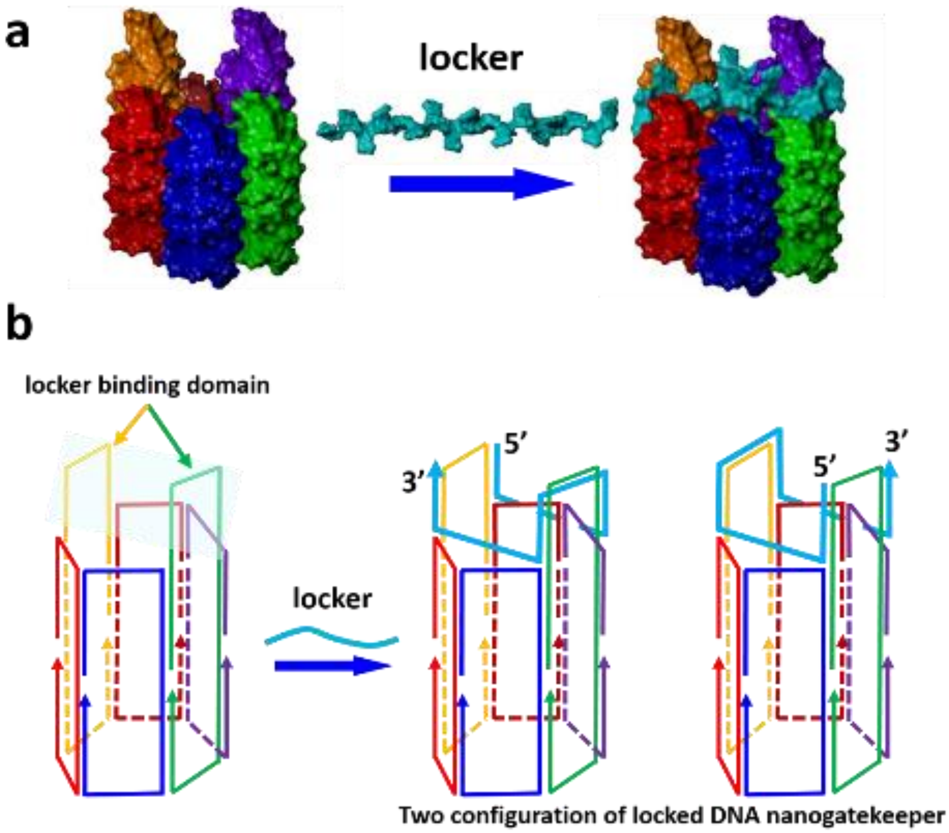

Supplementary Figure 8. Principle of the locker binding to the top region of nanogatekeeper. (a) Structural 3D model to illustrate that the locker hybridizes with the top region of nanogatekeeper, thereby plugging the nanogatekeeper forming the closed state. (b) Schematic diagram illustrating two possible configurations of locker binding to the top region of nanogatekeeper. For the fluorescence study, the fluorescent dye was modified at the 5' -end of the locker.

Circular dichroism (CD) spectroscopy analysis

To investigate the structural change of the locker with different concentrations of ATP, 200 μL samples were carried out for CD detection. The signal shows the configuration change of complex of locker with ATP at different concentration.

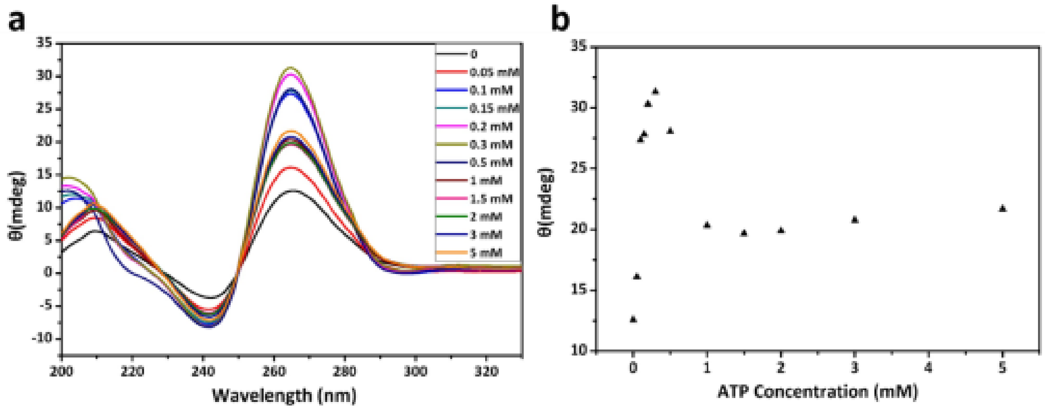

Supplementary Figure 9. Circular dichroism spectroscopy analysis for the configurational changes of lock strand in different concentrations of ATP: 0, 0.05, 0.1, 0.15, 0.2, 0.3, 0.5, 1, 1.5, 2, 3 and 5 mM. (a) CD spectra for 10 μM lock strand mixed with ATP at room temperature for 30 min. (b) θ value at 265 nm in previous different concentration of ATP.

3b. DNA nanogatekeeper in response to addition of ATP

To quantitatively analyze the response of closed nanogatekeeper to ATP, nanogatekeeper was labeled with two BHQ-1 molecules, while the lock strand was labeled with FAM at the strand's end. (See Supplementary Fig. S3 for the position of BHQ-1 modification in this DNA nanostructure). Then different concentrations of ATP were incubated with a solution of c-DNGK to study ATP response.

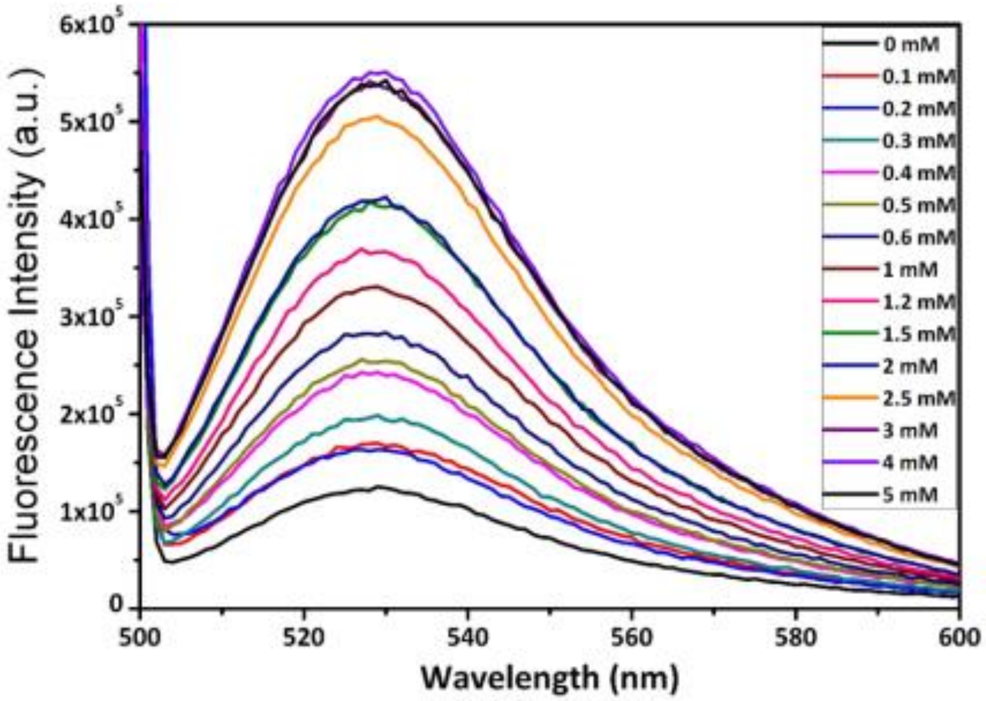

Supplementary Figure 10. Fluorescence intensity shows the effect of adding various concentrations of ATP. Fluorescence spectra of fluorescent dye-modified c-DNGK (1 μM) in response to the following different concentrations of ATP: 0, 0.1, 0.2, 0.3 0.4, 0.5, 0.6, 1, 1.2, 1.5, 2, 2.5, 3, 4 and 5 mM. λex=492 nm, bandpass=5 nm.

4. Microcompartment preparation

4a. Preparation of cell-mimicking giant membrane vesicles

Cell-mimicking giant membrane vesicles were obtained from detached HeLa cells. HeLa cells were obtained from ATCC (American Type Culture Collection, Manassas, VA, USA) and cultured in RPMI-1640 cell medium (Life Technologies, USA) supplemented with 10% fetal bovine serum (FBS, Gibco) and 1% penicillin (100 U/mL) -streptomycin (100 μg/mL, Life Technologies, USA) in a cell culture incubator at 37 °C with 5% CO_2 atmosphere. Cell density was determined using a hemocytometer. For adherent HeLa cells, short-term (30 s to 1 min) trypsin treatment was adopted to dissociate cells from the culture flask or dish. Giant membrane vesicles were derived from HeLa cells as previously reported. Briefly, HeLa cells were washed with DPBS four times after 48 h growth and then incubated in phenol red-free RPMI 1640 culture medium containing carboxylfullerenes for 4 hours at 37 °C. After removing cell medium, cells were washed with DPBS four times and added to 4 mL of RPMI 1640 culture medium (1×, without phenol red). Then, the adherent cells were irradiated under white light for another 4 hours. After overnight incubation, micron-scale giant membrane vesicles were suspended in the supernatant solution, and the collected supernatant fluid was used as prepared.

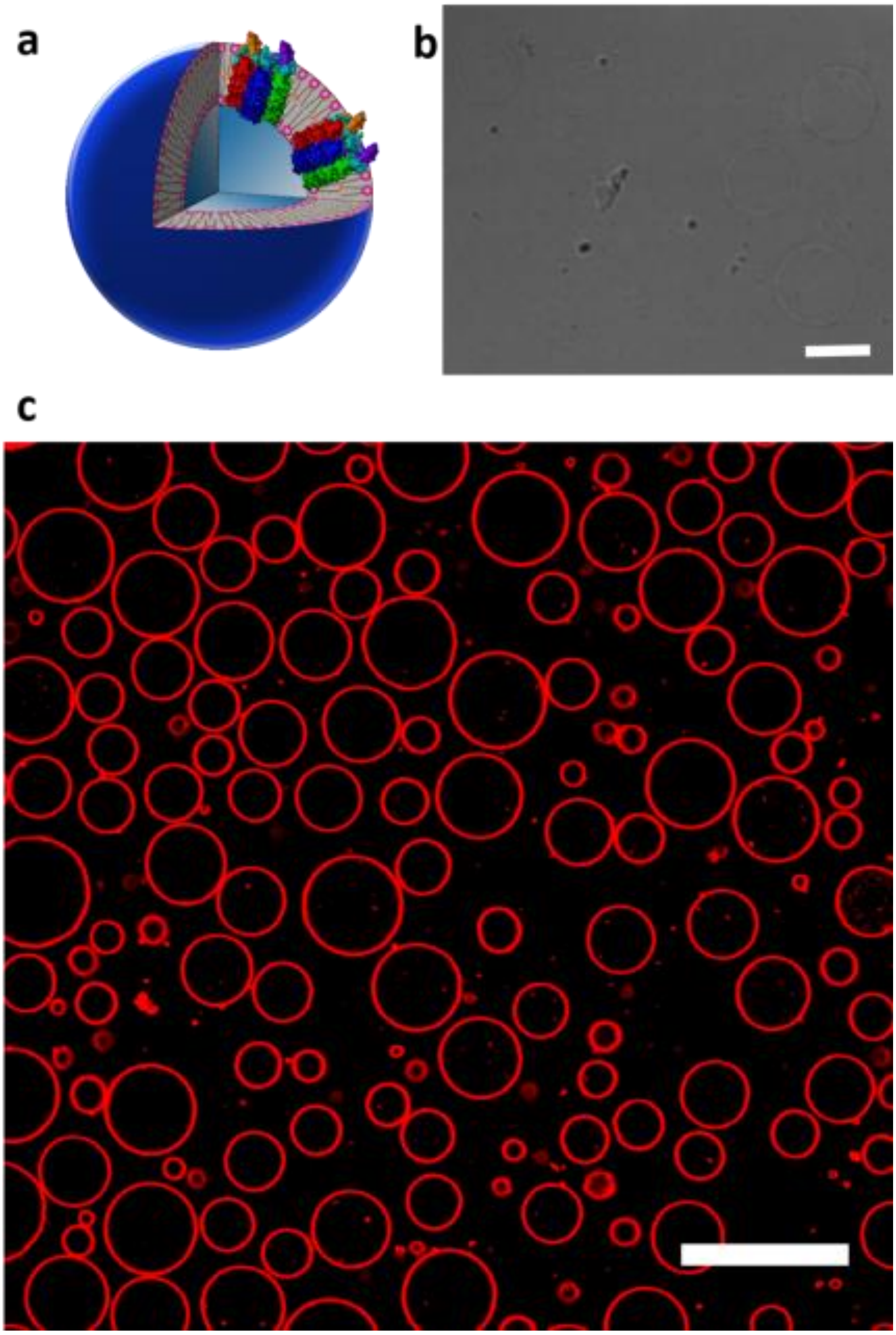

Supplementary Figure 11. Characterization of cell-mimicking giant membrane vesicles. (a) Schematic illustration of chol-c-nanogatekeeper (cholesterol-tethered nanogatekeeper in the closed state) anchored on the biomembranes of giant membrane vesicles. (b) Optical visualization of micro-scale giant membrane vesicles in bright-field. Scale bar, 15 μm. (c) Large field of giant membrane vesicles stained with FM 4-64 dye, a red fluorescence which showed the high yield of our cell-mimicking giant membrane vesicles. Scale bar: 40 μm.

4b. Preparation of giant unilamellar vesicle

A gentle hydration method was employed to generate a giant unilamellar vesicle, a phospholipid vesicle, which served as a general model of chemical synthesis of giant vesicles. A thin, dry film of phosphatidylcholines or lecithin was deposited from a 3 mL chloroform solution on the glass surface at the bottom of a flat-bottomed 500 mL flask. Before hydration, the lipid film was carefully dried. Hydration of the film was initiated by 3-4 mL circulating water through the flask overnight. The water was gently poured down the side of the flask.

5. Study of nanogatekeeper on cell-mimicking membrane vesicles

5a. Dynamic behavior of nanogatekeeper on cell-mimicking membrane vesicles

To demonstrate the dynamic behavior of the nanogatekeeper on cell-mimicking membrane, the mobility of chol-DNGK within the cell-mimicking membrane environment was studied using fluorescence recovery after photobleaching (FRAP). Interestingly, DNA nanostructures moved freely on the membrane of our giant vesicles. Recovery of fluorescence intensity was evident, indicating that the nanogatekeeper s are mobile and able to diffuse in and out of the bleached spot on a time scale comparable to that of the control fluorescent lipid molecules. These results show that this amphiphilic DNA nanostructure readily binds to the giant vesicles and undergoes Brownian motion on the membrane surface.

We chose CLSM to visualize the behavior of DNA nanostructures on giant membrane vesicles. Typically, a 500 μL solution of giant membrane vesicles was placed inside a 15 mm confocal dish and incubated with chol-DNGK at 37 °C for 30 min. After incubation, giant membrane vesicles were directly observed by CLSM imaging using the FV1000 confocal microscope (Olympus). Confocal fluorescence images were collected with an objective lens (60×, UplanApo N.A. 1.35, Olympus) with 30% laser intensity for real-time observation experiments. And the laser intensity for photobleaching is 100%, the area of bottom half of GPMV was selected.

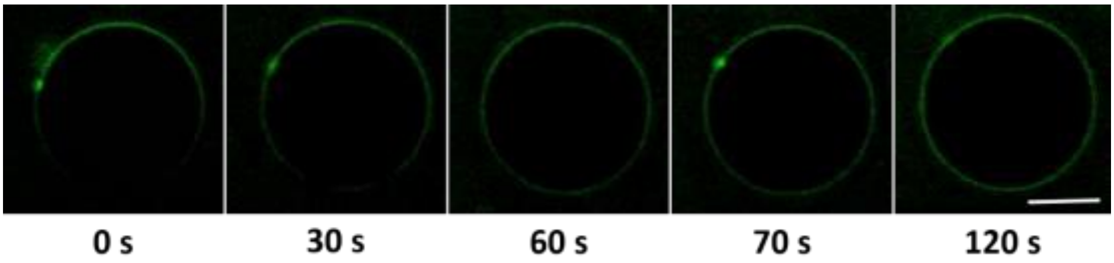

Supplementary Figure 12. Acceptor photobleaching experiment demonstrates the dynamics of the giant membrane vesicles. Alexa Fluor 488-labeled chol-c-DNGK (12.5 μM, 40 μL) incubated with 400 μL giant membrane vesicles at 37 °C for 30 min. Diffusion of DNA nanostructure through the membrane of giant vesicles. Scale bar: 5 μm.

5b. Studies of nanogatekeeper on giant unilamellar vesicles

To demonstrate the platform of employing DNA nanostructure to engineer a membrane compartment, we also studied a giant unilamellar vesicle (GUV) as a common synthetic membrane model.

For flow cytometric analysis, GUVs were suspended on the BD FACSVerse™ flow cytometer by counting 100,000 events. Initially, 1.2 µL 500 µM FluoZin™-3 indicator was incubated with 200 µL giant unilamellar vesicles at 37 °C for 2 h. Then after incubation with 40 µL of 2.5 µM chol-DNGK for 30 min, 3.2 µL of 12.5 mM Zinc ion was added.

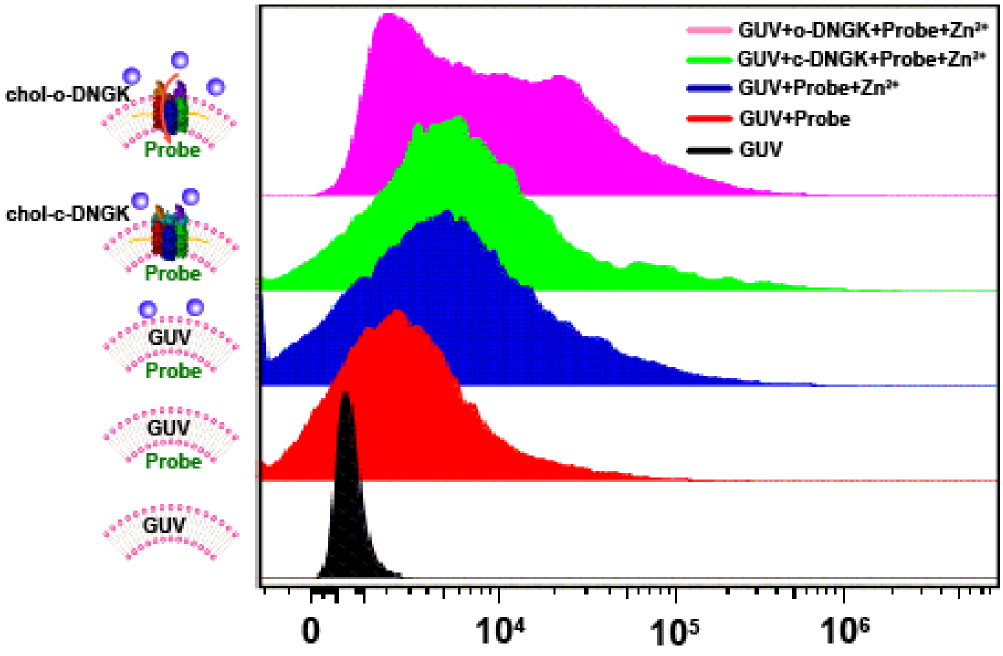

Supplementary Figure 13. Flow cytometry study of DNA nanogatekeeper anchored on GUV membrane. Probe of FluoZin™-3 was used to selectively signal zinc ion.

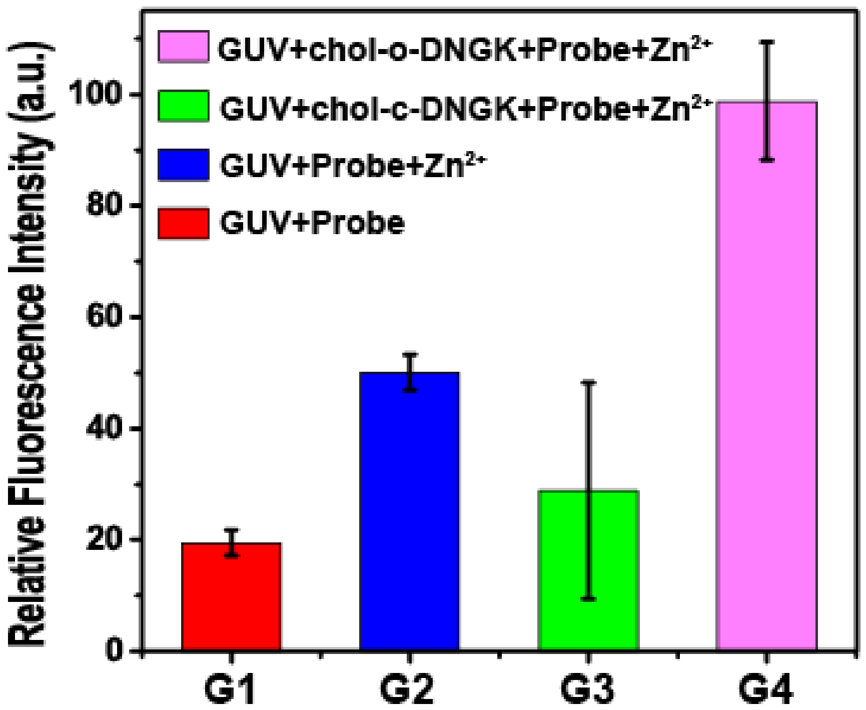

Supplementary Figure 14. Statistical evaluation with flow cytometry of nanogatekeeper anchoring on GUV. Error bars show the standard deviation of three independent experiments.

5c. Ca^{2+} channel mimicry of nanogatekeeper

Together with using Zn^{2+} fluorescent probe (FluoZin™-3 indicator) to investigate ion channel mimicry of the DNA nanogatekeeper, we alternately chose calcium ion as the model to investigate the efficiency of Ca^{2+}. The calcium probe of calcein could barely cross the membrane to aggregate in the chamber of our giant vesicles; therefore, we chose the acetoxymethyl (AM) ester derivatives of calcein AM. 5 µL calcein AM (in DMSO solution) were added into 400 µL solution of giant membrane vesicles, followed by incubation with nanogatekeeper for 30 min at 37 °C. 3 mM of ATP was added to react for 5 min to open the gate.

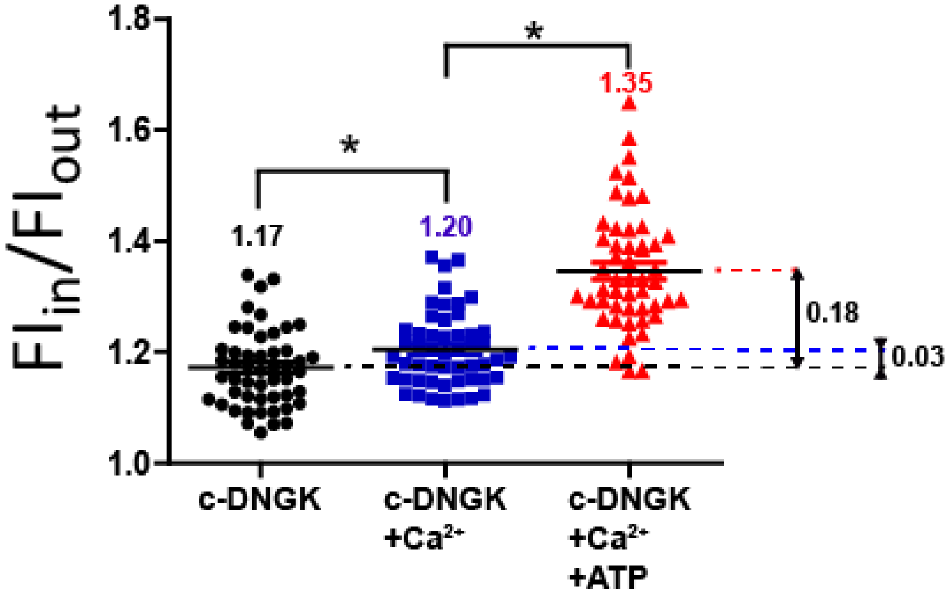

Supplementary Figure 15. The efficiency of nanogatekeeper to ions. Fluorescence change response to nanogatekeeper. First group: Calcein-AM entrapped in chamber of giant membrane vesicles and 400 µL GVs incubated with Calcein-AM overnight; Second group: previous addition of Ca^{2+}; Third group: previous addition of ATP. Average ionic leakage for c-DNGK = (1.20-1.17)/(1.35-1.17)=16.7%. Statistical sample population of giant membrane vesicles =50, *$P<0.05$, Student's *t*-test, using GraphPad Prism 5.

6. In solution construction of DNA networks

The confined DNA-based molecular networks were designed with two parts. The main molecular network is driven by zinc ion which activates a DNA circuit for mimetic target degradation. Another part is the responsive feedback DNA pathway to return the membrane-anchored nanogatekeeper to the closed state.

Supplementary Table 6. DNA sequences of the confined artificial molecular signaling network

Name	DNA sequences (5'-3')
E	TAAAATTTATTCTCCGAGCCGGTCGAAATAGTGAGT
E-Q	TAAAATTTATTCTCCGAGCCGGTCGAAATAGTGAGT-BHQ1
Sub-I	ACTCACTAT *rA* GGAATA-AATTTTAGGCCC
FAM-Sub-I	FAM-ACTCACTAT *rA* GGAATA-AATTTTAGGCCC
F (4-3-2)	AATTTTAGGCCC-GGTC-AACATTTCTCCA ACTAACTTACGT
F-Cy5	AATTTTAGGCCC-GGTC-AACATTTCTCCA ACTAACTTACGT-Cy5
S-I (5-4)	GGAATA-AATTTTAGGCCC
W (4-3)	AATTTTAGGCCC-GGTC
S-II (2-1)	AACATTTCTCCA ACTAACTTACGT-CAGCTGTTTCAGCTG
M1 (2*-3*-4*-5*)	ACGTAAGTTAGTTGGAGAAATGTT-GACC-GGGCCTAAAATT-TATTCC
M-Cy3	Cy3-ACGTAAGTTAGTTGGAGAAATGTT-GACC-GGGCCTAAAATT-TATTCC
T	**CAGCTG**AAA**CAGCTG**ACGATTTCCGCT**CAGCTG**AAA**CAGCTG**AC GTAAGTTAGT
Ligation	ACGACAGCTGAAACAGCTGGCTC
Circle	*PO_4-* TCAGCTGTCGTAGGACTTAAACTGAGCGGAAATCGTCAGAAAAGG CAAGATAGAGCCAGCTGTT
SG	GACGCACGACTGGTTGGTT-GACACTAGCTGGATCTGAGGATTAGTA
I	ACTAATCCTCAGATCCAGCTAGTGTC-AATGGAAGGTA GTCGTGC

Blocker 1	***ACTAATC***CTCAGATC***CAGCTAG***TGTCACTA***ATCCTCA***GATCCAGC***TAGTGTC***
SG-Q	GACGCACGACTGGTTGGTTGACACTAGCTGGATCTGAGGATTAGTA-BHQ3
Cy5-Blocker 1	Cy5-***ACTAATC***CTCAGATC***CAGCTAG***TGTCACTA***ATCCTCA***GATCCAGC***TAGTGTC***
W*	GACCGGGCCTAAAATT
Blocker 2	***AGGCCCG***GTCTTTTT***CTGTAGG***TTTTTCCC***AGGCCCG***GTCTTTT***CTGTAGG***
W*-Q	GACCGGGCCTAAAATT-BHQ3
Blocker2-F	Cy5-***AGGCCCG***GTCTTTTT***CTGTAGG***TTTTTCCC***AGGCCCG***GTCTTTT***CTGTAGG***

Blocker strands, as indicated by letters in bold and italics, denote binding bases.

rA denotes the ribonucleotide adenine.

Red letters represent the modification including FAM, Cy5, BHQ 1, BHQ 3 (black hole quencher-3) and $-PO_4$.

6a. Module of ion-mediated catalysis

A highly efficient Zn^{2+}-dependent deoxyribozyme (DNAzyme, 17E) shares a catalytic core similar to that of the 8-17 deoxyribozyme motif. Importantly, the arm of 17E deoxyribozyme/substrate complex can be designed. Based on a similar structure, we redesigned this catalytic nucleic acid for the first activation step.

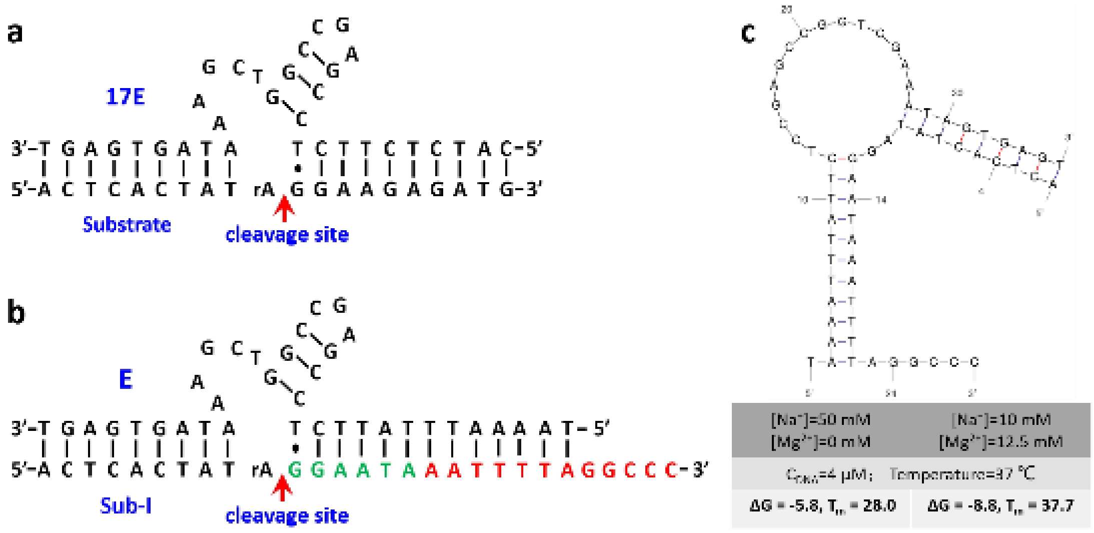

Supplementary Figure 16. Sequence design of DNAzyme-based activation. (a) The reported proposed secondary structure of 17E deoxyribozyme-substrate complex. (b) Our design based on 17E DNAzyme. The red arrow indicates the cleavage site. The zinc ion-mediated activation step was designed based on this structure. (C) Configuration of the hybridization of E and Sub-I with Mfold. Free energy is in kcal/mol, and melting temperature is in °C.

Gel electrophoresis for catalysis

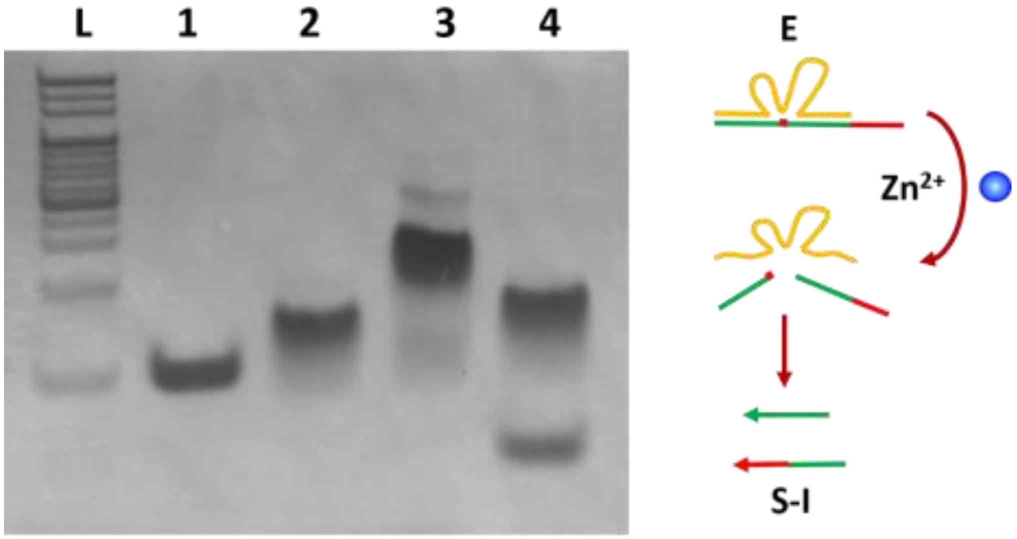

Supplementary Figure 17. PAGE (12%) analysis of DNAzyme. Effect of our redesigned Zn^{2+}-dependent DNAzyme (E) cleaved substrate (Sub-I) strand producing initial signal (S-I) strand in 50 mM Na-HEPES buffer. Lane 1: 4 μM Sub-I strand. Lane 2: 4 μM E strand. Lane 3: the hybrid of E strand and Sub-I strand (E/Sub-I) by overnight annealing. Lane 4: E/Sub-I with 2 mM Zn^{2+} incubated at 37 °C for 30 min.

Titration curve of ion-mediated catalysis

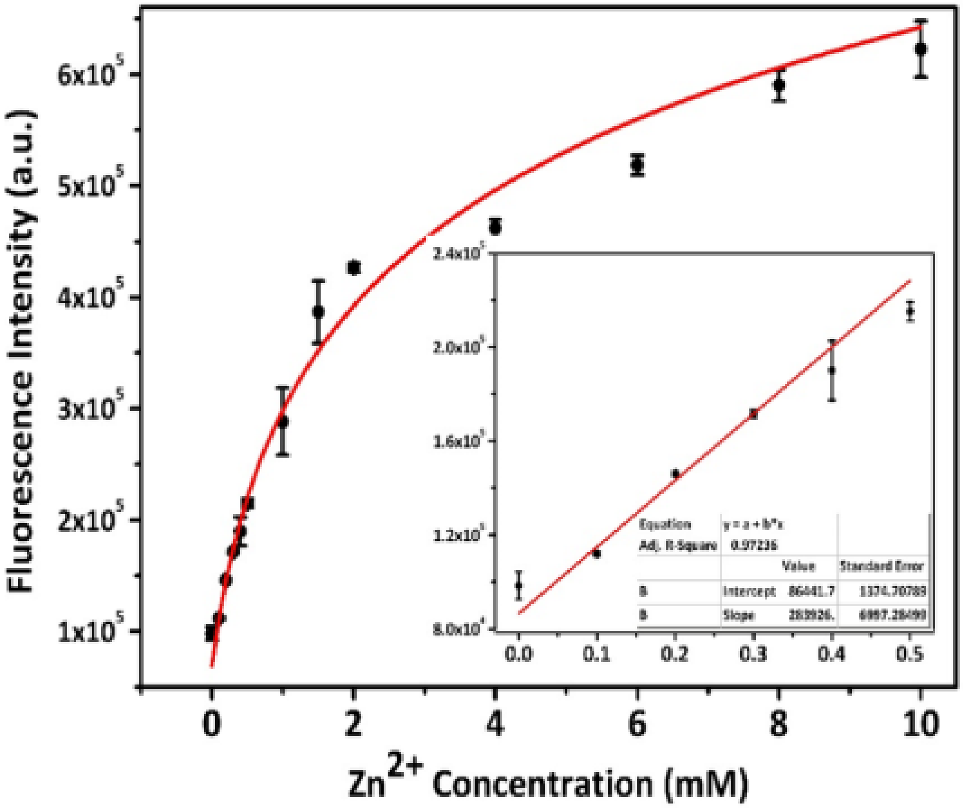

Supplementary Figure 18. Fluorescent change of 100 nM Zn^{2+}-dependent DNAzyme in various concentrations of zinc ion (200 μL, 50 mM HEPES, 1.5 M NaCl, pH=7.0): 0, 0.1, 0.2, 0.3, 0.4, 0.5, 1, 1.5, 2, 4, 6, 8 and 10 mM. Linear relationship between FI (fluorescence intensity) and low concentrations of Zn^{2+} (r^2=0.97). The E strand was labeled with BHQ 1 (quencher), while Sub-I strand was labeled with FAM (fluorophore). The complex of E/Sub-I incubated with Zn^{2+} at 37 °C for 30 min, λex=492 nM, λem=520 nM, bandpass=5 nm (means±3).

6b. DNA circuit signaling transduction

This module is based on entropy-driven DNA circuitry. In the absence of initial signal (S-I), the signaling is inactivated for ΔG=0. However, in the presence of S-I, the secondary signal is generated and amplified.

Design of DNA circuit signaling transduction

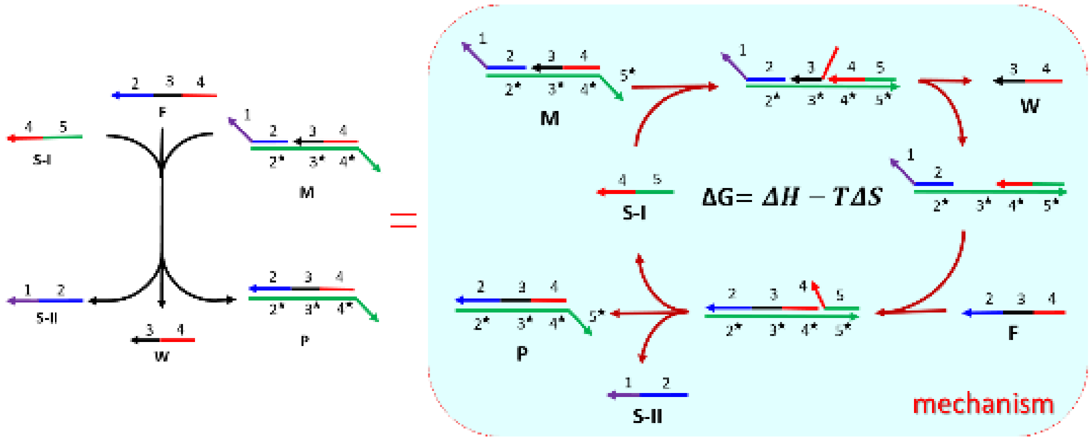

Supplementary Figure 19. Principle of entropy-driven DNA circuit. The initial signal strand (S-I) is released from the ion-mediated activation step, followed by mimetic signal transduction, which produces the secondary signal strand (S-II). S-II acts as the signal mediating downstream target degradation. Domain N* is completely complementary to domain N, and the details of each domain are shown in Supplementary Table 7.

Supplementary Table 7. Design of entropy-driven DNA circuit

Dom.	Sequences (5'-3')	Dom.	Sequence (5'-3')	Length
1	CAGCTGTTTCAGCTG	1*	CAGCTGAAACAGCTG	15
2	AACATTTCTCCAACTAAC TTACGT	2*	ACGTAAGTTAGTTGGAGA AATGTT	24
3	GGTC	3*	GACC	4
4	AATTTTAGGCCC	4*	GGGCCTAAAATT	12
5	GGAATA	5*	TATTCC	6

Strand domain of N* is completely complementary to the domain of strand N.

Thermodynamics calculation of DNA circuit signaling transduction

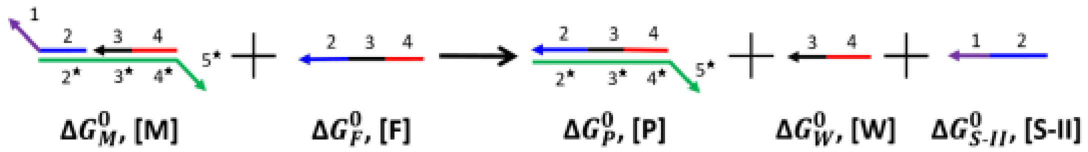

Supplementary Figure 20. Reaction equation of signaling in the presence of initial signal strand (S-I) strand with thermodynamic parameters.

As shown in Supplementary Figure 20, the Gibbs free energy change (ΔG) for this reaction in dilute solutions is

$$\Delta G = \Delta H - T \cdot \Delta S \quad \text{(Eq.1)}$$

where ΔH is the system enthalpy change, ΔS is the entropy change, and T is the thermodynamic temperature. The total number of base pairs in the reactants and products is unchanged, giving ΔH ≈ 0. Hence, the reaction is driven forward thermodynamically by the entropic gain of the liberated molecules, and the driving force, at any moment, is TΔS.

The final concentration of all species in this entropy-driven system can now be estimated. According to Van 't Hoff equation,

$$\Delta G = \Delta G_{\mathrm{P}}^{0} + \Delta G_{W}^{0} + \Delta G_{S-II}^{0} - \Delta G_{\mathrm{M}}^{0} - \Delta G_{F}^{0} + RT \ln Q \quad \text{(Eq.2)}$$

where Q = [([P]/c^{o})([W]/c^{o})([S-II]/c^{0})]/[([M]/c^{0})([F]/c^{0})] is the reaction quotient relative to standard conditions, and $\Delta G^{0}{}_{X}$ is the standard free energy of species X at standard conditions, which herein are represented by our TAE buffer condition with 12.5 mM Mg^{2+}, 25 °C, and c^{0}=1 M.

ΔG_{X}^{0} can be calculated by using open source software, such as Mfold and NUPACK, giving

$$\Delta G_{\mathrm{P}}^{0} + \Delta G_{W}^{0} + \Delta G_{S-II}^{0} - \Delta G_{M}^{0} - \Delta G_{F}^{0} = -1.34(\mathrm{Kcal}/mol)\ . \quad \text{(Eq.3)}$$

When the reaction reaches equilibrium, which means ΔG=0, according to Eq. 2 and 3, we can determine that Q=9.61.

According to the former equation, we know that Q = [([R]/c^{o})([P]/c^{o})([W]/c^{0})]/[([B]/c^{0})([F]/c^{0})] and c^{0}=1 M. For a system in which the initial concentration of both M and F is 10 nM and the final concentration of P is x nM, we can give the following equation:

$$(10^{-9} \cdot x)^{3}/[10^{-9} \cdot (10 - x) \cdot 10^{-9} \cdot (10 - x)] = 9.61 \quad \text{(Eq.4)}$$

Using the bisection method, we can estimate x to be between 9.999 and 9.9999 nM, which

means a potential efficiency of this reaction is more than 99.99% without regard for the reaction time.

Gel electrophoresis analysis for DNA circuit signaling transduction

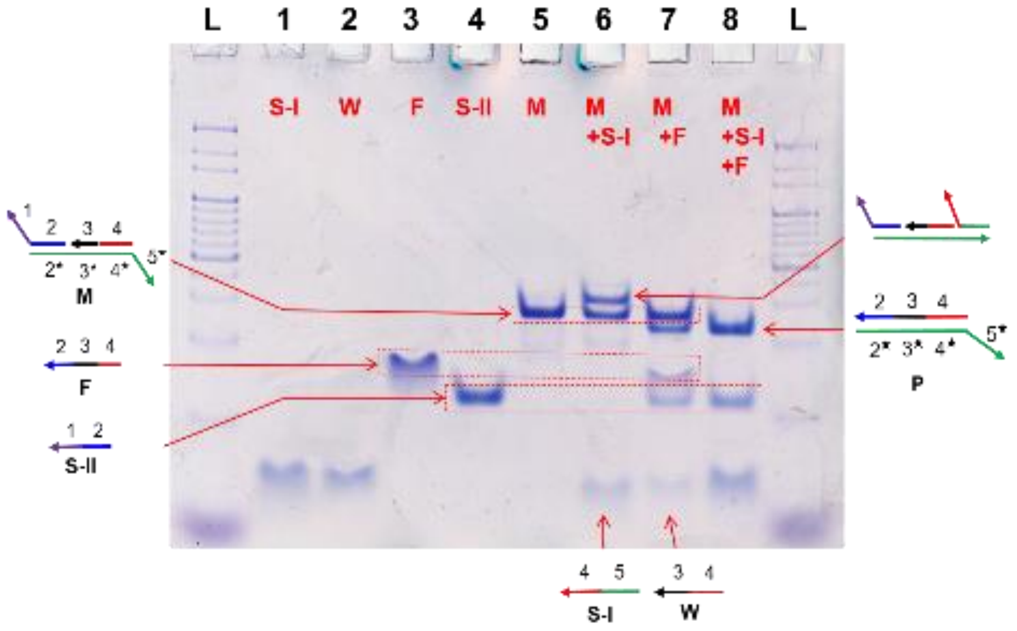

Supplementary Figure 21. PAGE (12%) analysis for module of entropy-driven DNA circuit signaling, running at 110 V for 120 min in an ice-water bath. All sample volumes were 10 µL. Lane 1: 1.5 µM S-I strand; Lane 2: 1.5 µM W strand; Lane 3: 1.5 µM F strand; Lane 4: 1.5 µM S-II strand; Lane 5: 1.5 µM M strand; lane 6: incubating 1.5 µM M strand and 1.5 µM S-I strand for 30 min; lane 7: incubating 1.5 µM M strand and 1.5 µM F strand for 30 min; lane 8: incubating 1.5 µM M strand, 1.5 µM S-I strand and 1.5 µM F strand for 30 min. Lane L: 20 bp DNA ladder consisting of double strands of DNA with length increase in 20-bp steps. All DNA bands were stained with Stains-All.

Qualitative fluorescence analysis of DNA circuit signaling transduction

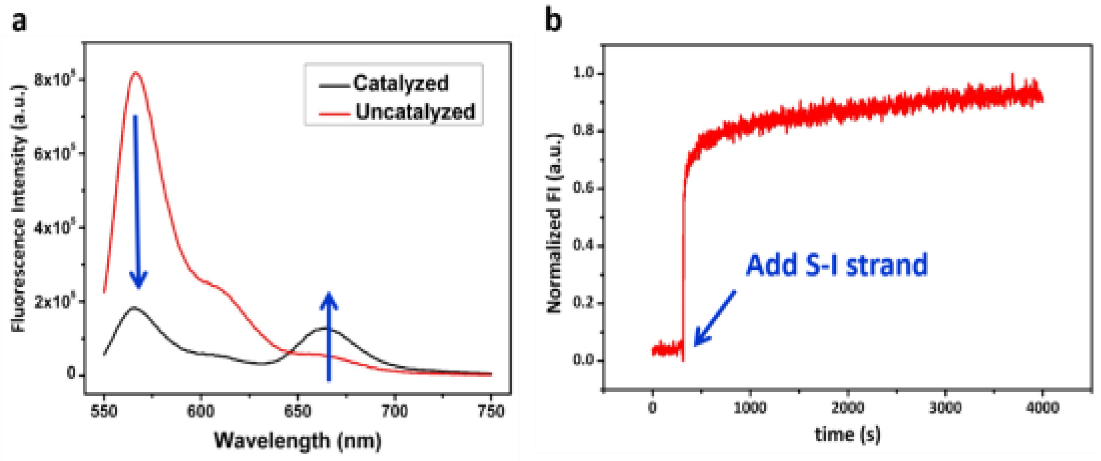

Supplementary Figure 22. Fluorescence change of entropy-driven DNA cascade reaction. The tested sample volume was 200 μL. (a) red line: complex M (100 nM M1+100 nM S-II +100 nM W) with 300 nM F; black line: previous samples addition of 100 nM S-I. (b) Time course study of entropy-driven DNA cascade reaction in the presence of strands S-I and F. The results showed that the DNA circuit reaction was driven by adding the catalytic S-I strand. λex=525 nm, λem=665 nm, bandpass=2 nm.

Quantitative fluorescence analysis of DNA circuit signaling transduction

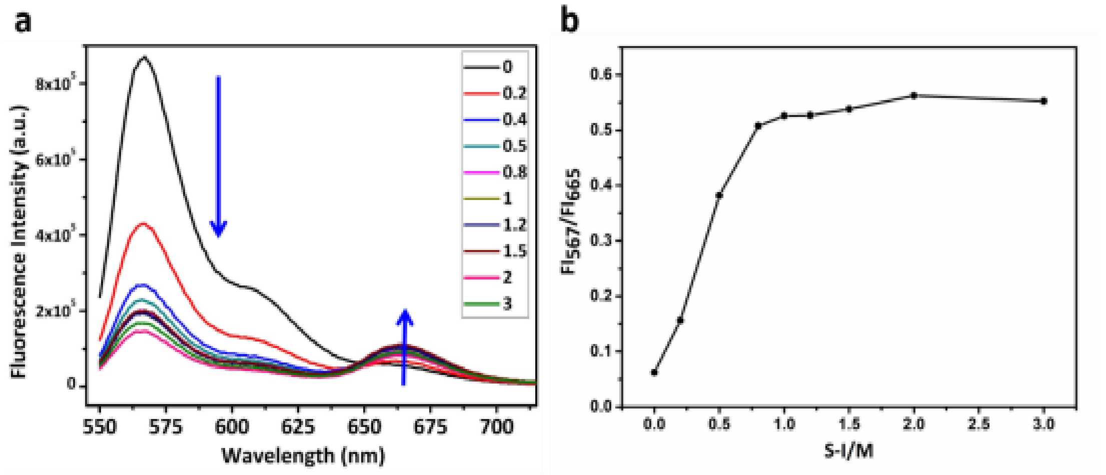

Supplementary Figure 23. (a) Fluorescence change of different concentration of S-I added to a solution of 100 nM M and 300 nM F. The right column shows the concentration ratio of S-I to M (0, 0.2, 0.4, 0.5, 0.8, 1, 1.2, 1.5, 2 and 3), λex=525 nm. (b) Fluorescence intensity ratio (FI_{567}/FI_{665}) showing the effect of adding different concentration of S-I strand. All bandpass=5 nm.

6c. Endonuclease-recognized target degradation

In this module, we used Pvu II, a restriction endonuclease which recognizes CAG^CTG sites, in double strand for target strand degradation.

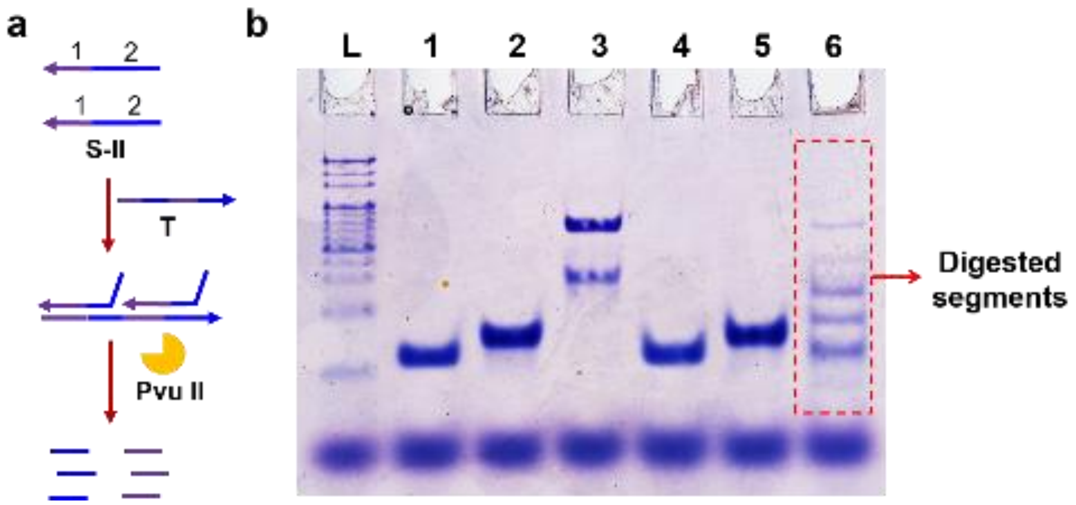

Supplementary Figure 24. Endonuclease-recognized target degradation module. (a) Schematic illustration of enzyme-mediated degradation of target strands. (b) 12% PAGE analysis of DNA complex (S-II+T) cleavage by restriction endonuclease Pvu II, running at 110 V for 60 min in an ice-water bath. Lane 1: 2 μM S-II strand; lane 2: 2 μM T strand; lane 3: incubating 2 μM S-II strand and 1 μM T strand for 30 min; lane 4-6: samples from lane1-3 with addition of 0.5 U Pvu II, respectively, followed by incubation for enzymatic degradation at 37 °C for 10 min. Bands, as shown in the red box (lane 6), were digested into segment. Lane L: 20 bp DNA ladder consisting of double strands of DNA with length increase in 20-bp steps. All DNA bands were stained with Stains-All and then imaged with the Microtek's Bio-6000 scanner.

6d. Characterization of integrated modules

Next, the integration of individual modules was investigated. To demonstrate the feasibility of our signaling network, we gradually integrated the above individual modules and studied the resultant integration with fluorescence and gel electrophoresis.

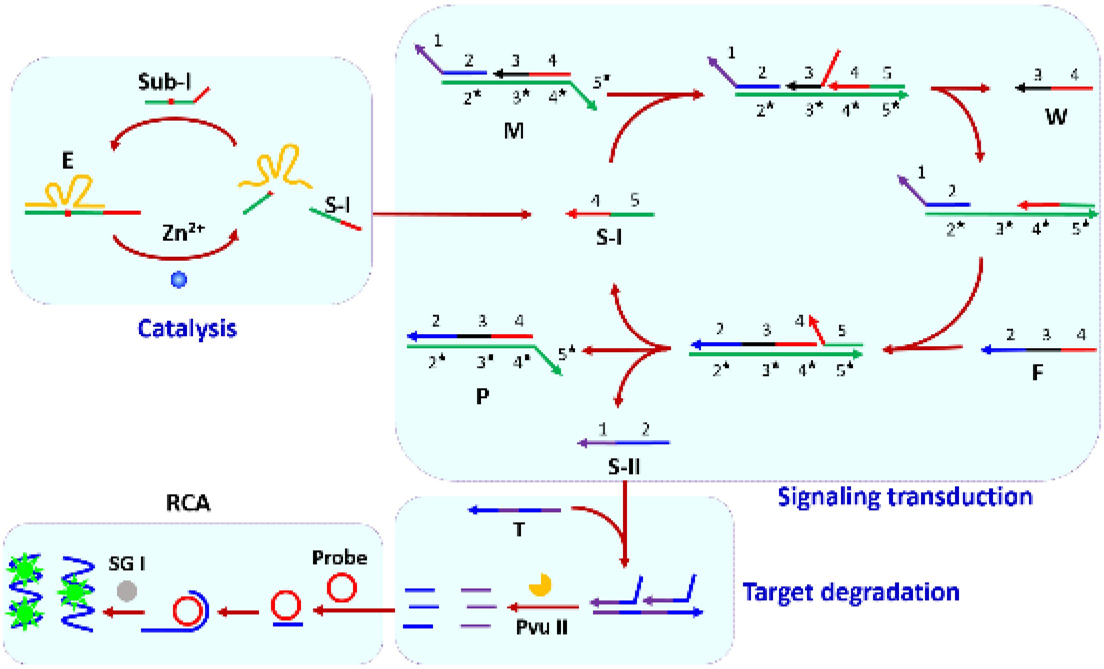

Supplementary Figure 25. Schematic representation of signaling network without feedback pathway, but including the modules of catalysis, signaling transduction and target degradation. Finally, the signal was detected by RCA reaction. Theoretically, modules 1 and 2, as well as RCA, are DNA amplification reactions.

Fluorescence intensity analysis for integrated modules of catalysis and signaling transduction

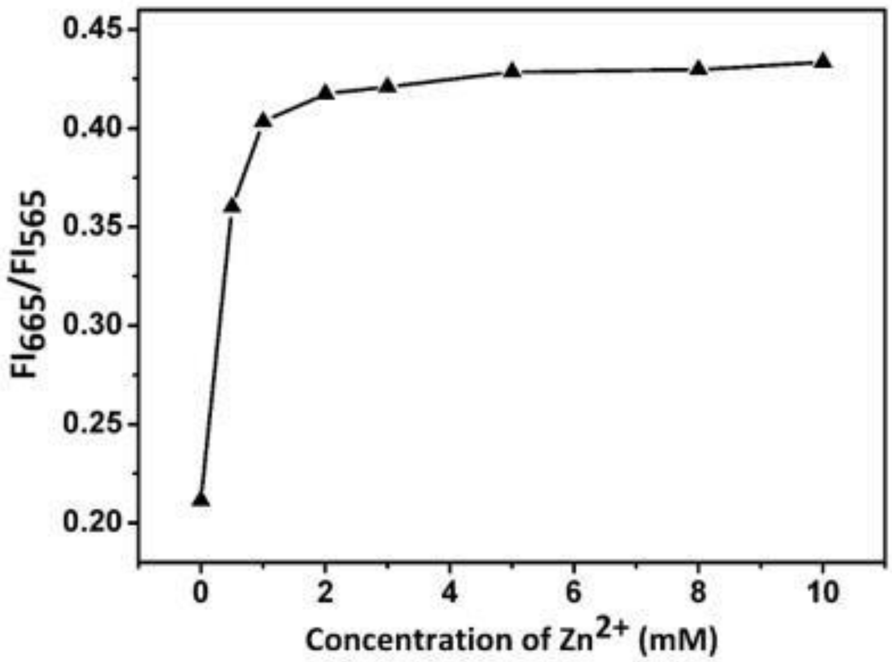

Supplementary Figure 26. Fluorescence change study to investigate the integrated modules of 1 and 2. Different concentrations of zinc ion (0, 0.5, 1, 2, 3, 5, 8 and 10 mM) were added into reaction substrate mixtures of 300 nM E, 100 nM M, 300 nM F in TAE/Mg^{2+} buffer (40 mM Tris 12.5 mM Mg^{2+}, 1 mM EDTA, pH=7.4). λex=525 nm, bandpass=5 nm.

6e. Rolling circle amplification (RCA) to detect degradation products

Preparation of RCA probe

Circular DNA probe was ligated by ligation of 5′-phosphorylated circular DNA sequences with the help of additional ligation template by T4 DNA ligase. T4 DNA ligase catalyzes the formation of phosphodiester bonds between 5'-P and 3'-OH of circular DNA fragments. The ligation reaction was conducted in a volume of 10 μL. One μL of linear, 5′-phosphorylated circular probe DNA template (10 μM) was hybridized with 1 μL of ligation template (100 μM) in 1× T4 DNA ligase reaction buffer (400 mM Tris-HCl, 100 mM magnesium chloride, 100 mM Dithiothreitol, 5 mM ATP, pH 7.8) at 22 °C for 30 min before ligation. Ligation was performed at 22°C for 2 h by adding 1 U T4 ligase and then heating at 65 °C for 15 min to terminate the reaction. Next, 20 U Exonuclease I and 100 U Exonuclease III were added to digest the leftover ssDNA and dsDNA to yield closed circular DNA template. The enzymes were denatured by heating at 80 °C for 20 min. These prepared probes were then stored at -20 °C until use.

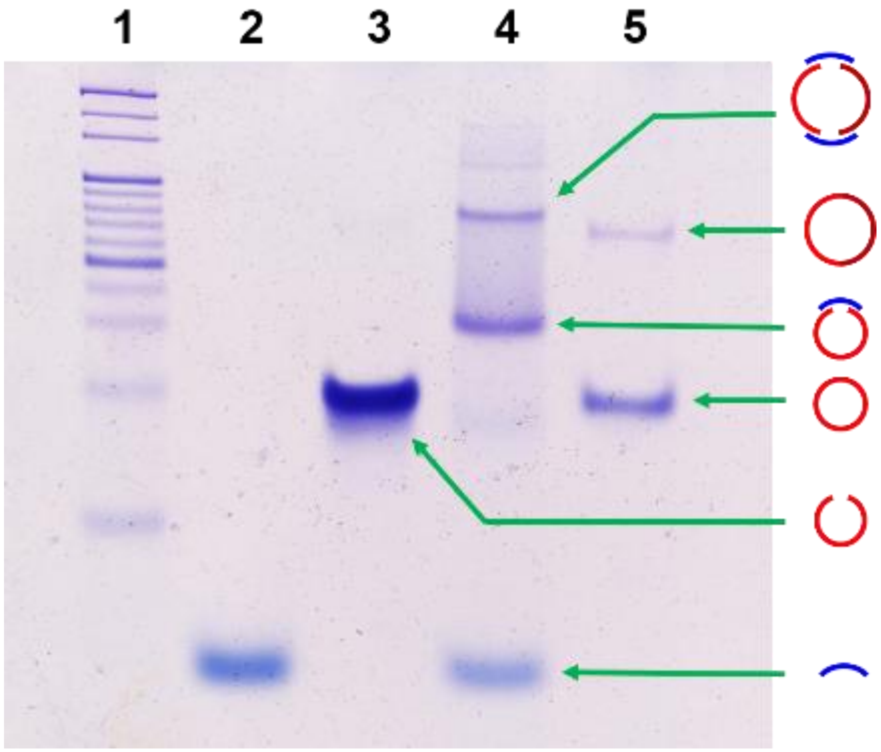

Supplementary Figure 27. PAGE (12%) result of RCA probe preparation, running at 110 V for 80 min in an ice-water bath. Lane 1: 20 bp DNA ladder. Lane 2: 4 μM ligation strand. Lane 3:4 μM circular strand. Lane 4: 2 μM circular strand and 4 μM ligation strand. Lane 5: previous sample with

the addition of 20 U Exo I and 100 U Exo III, followed by digestion at 37°C for 1 h.

Agarose gel electrophoresis detection for digested segments

For 3% agarose gel preparation, 1.2 g agarose powder was added to 40 mL 1× TAE/Mg^{2+} buffer and microwaved twice until agarose was completely dissolved. Then the agarose solution was placed at room temperature and left to cool for about 5 min, after which 3 μL of ethidium bromide (EB) was added to the solution. Before sample electrophoresis analysis, the gel was run in TAE/Mg^{2+} for 10 min. Finally, all samples were added, and the gel was run for at 110 V and imaged under Bio-Rad's ChemiDoc XRS System.

For RCA, the detected sample (digested segments of hybrid of T/S-II) was mixed with the prepared circular probe, 1 μL dNTP, 1 μL BSA, 1 μL buffer and 1 μL Phi 29, followed by incubation at 37 °C for 1.5 h.

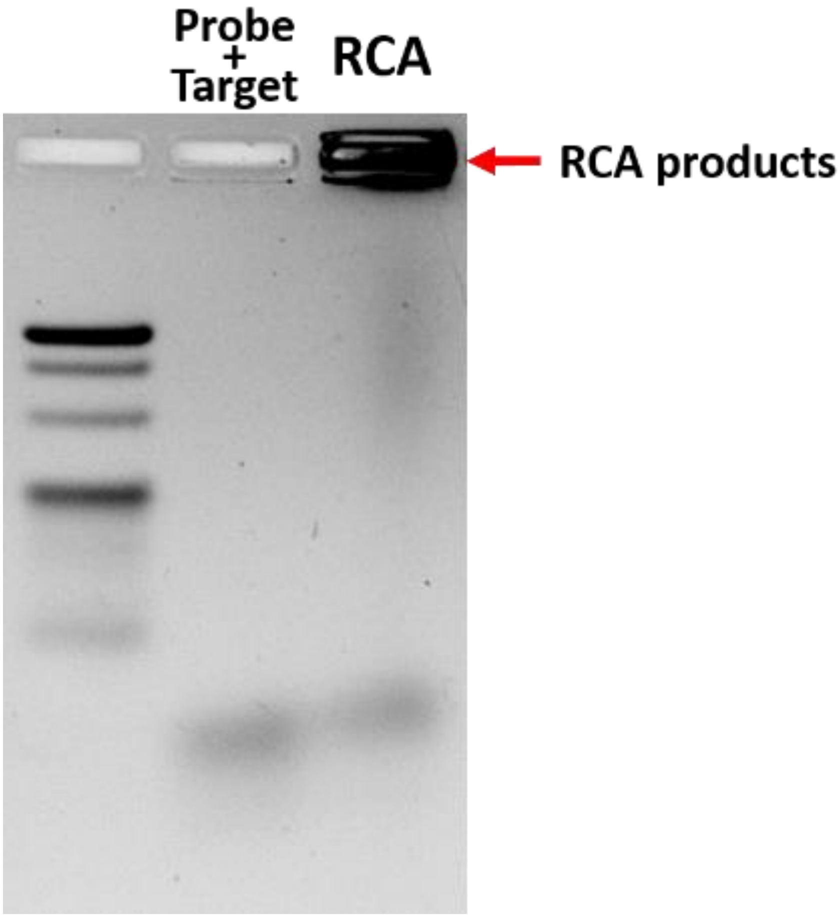

Supplementary Figure 28. Agarose gel electrophoresis (3%) results of RCA to detect target DNA. The mixture of circular probe and digested segments of hybrid of T/S-II were added. Then, the

previous final product was used to perform RCA. The results demonstrated the feasibility of employing RCA for detecting the digested product of complex of T/S-II.

Fluorescence analysis for RCA

In order to more visually observe the reaction, an optical method was used to monitor the rolling circle amplification product. RCA was performed on the following three groups, including buffer solution, uncatalyzed signaling network and catalyzed signaling network. Only the catalyzed signaling network could release the target strand (digested segments), followed by RCA. Finally, the compact product increased the fluorescence signal of SG-I.

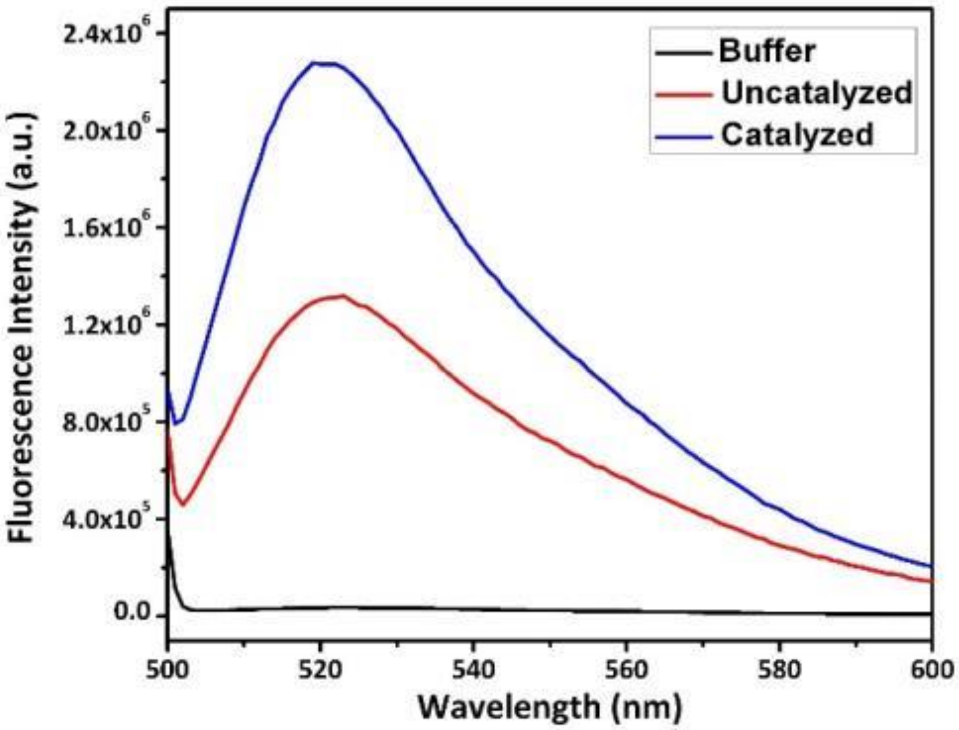

Supplementary Figure 29. Fluorescence intensity change after RCA. As the artificial molecular signaling network catalyzed, the degradation products were produced. Through RCA, these products could be detected. Black: SG-I in buffer solution. Red: uncatalyzed signaling network (in the absence of Zn^{2+}). Blue: catalyzed signaling network (in the presence of Zn^{2+}).

6f. Feedback pathway

Design of the nanogatekeeper for feedback response

As shown in Supplementary Figure 30, we redesigned the 6-helix bundle nanostructure for feedback response. The locker (green DNA strand) hybridizes with the top region of nanogatekeeper, while the blocker (black DNA strand) hybridizes with the bottom region of nanogatekeeper.

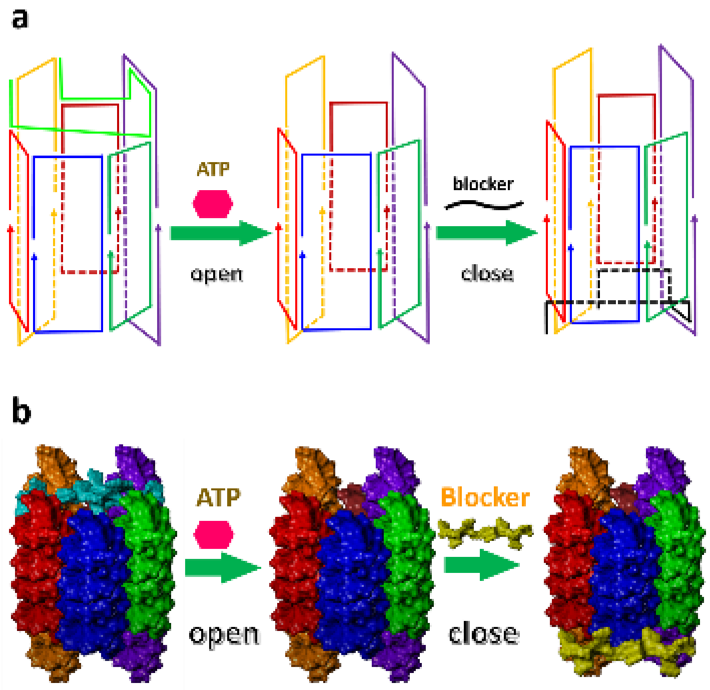

Supplementary Figure 30. Working principle of nanogatekeeper for triggered opening and responsive closing. (a) Illustration of DNA strand hybridization in different states. (b) 3D model corresponding to panel a.

Paralleled ion-activated feedback pathway

To design an ion-activated feedback pathway, a toehold-mediated DNA strand displacement reaction was created.

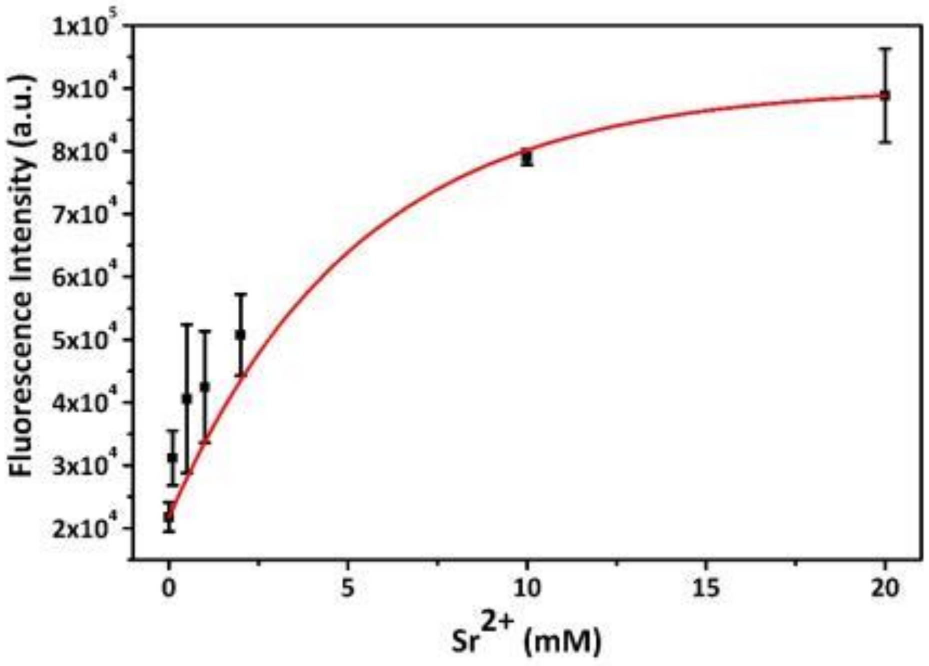

Supplementary Figure 31. Fluorescence change of 20 nM sample in different concentration of Sr^{2+}, including 0, 0.1, 0.5, 1, 2, 10 and 20 mM (means±3).

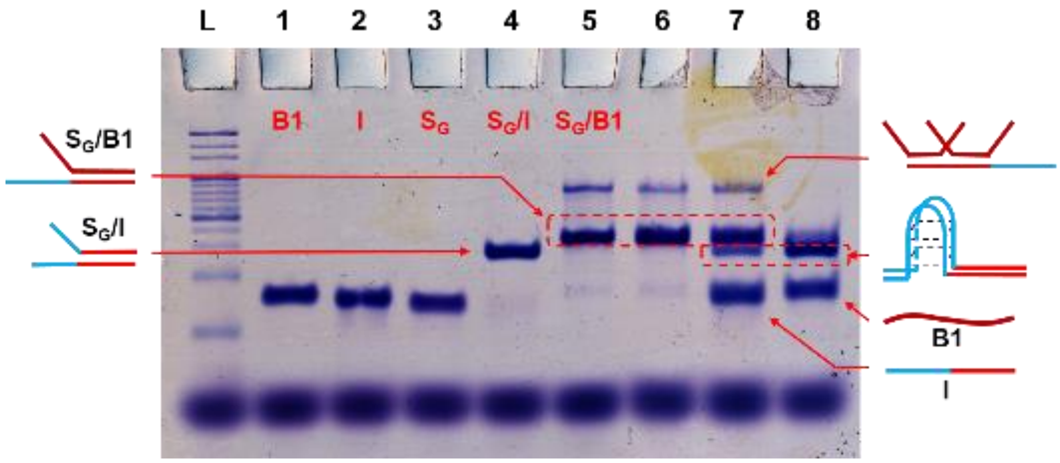

Supplementary Figure 32. PAGE (12%) for analysis of feedback pathway, running at 110 V for 60 min in an ice-water bath. Volume of all samples is 10 μL with Concentration of 2 μM. Lane L: 20bp DNA ladder; Lane 1: B1 strand; Lane 2: I strand; Lane 3: S_G strand; Lane 4: complex of S_G and I (S_G/I); Lane 5: complex of S_G and B1 (S_G/B1); Lane 6: previous sample with addition of Sr^{2+}; Lane 7: hybrids of S_G/B1 (sample in Lane 4) with addition of I strand (sample in Lane 1); Lane 8: Mixture of hybrid of S_G/B1, I and Sr^{2+}, showing that Sr^{2+} triggered the toe-hold mediated DNA strand displacement reaction.

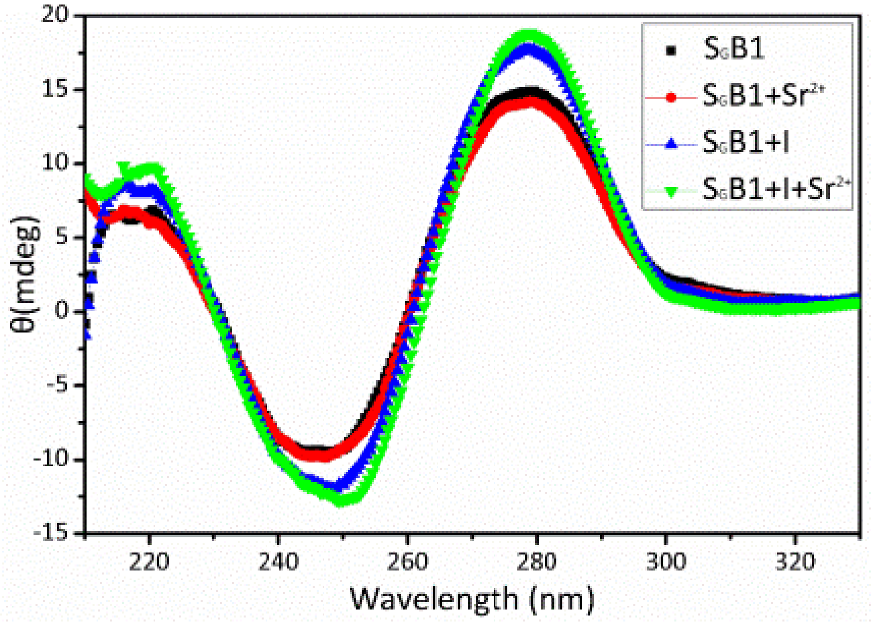

Supplementary Figure 33. Circular dichroism spectral analysis for the configuration changes of 200 μL 10 μM sample. A small shift can be observed in the catalytic sample group.

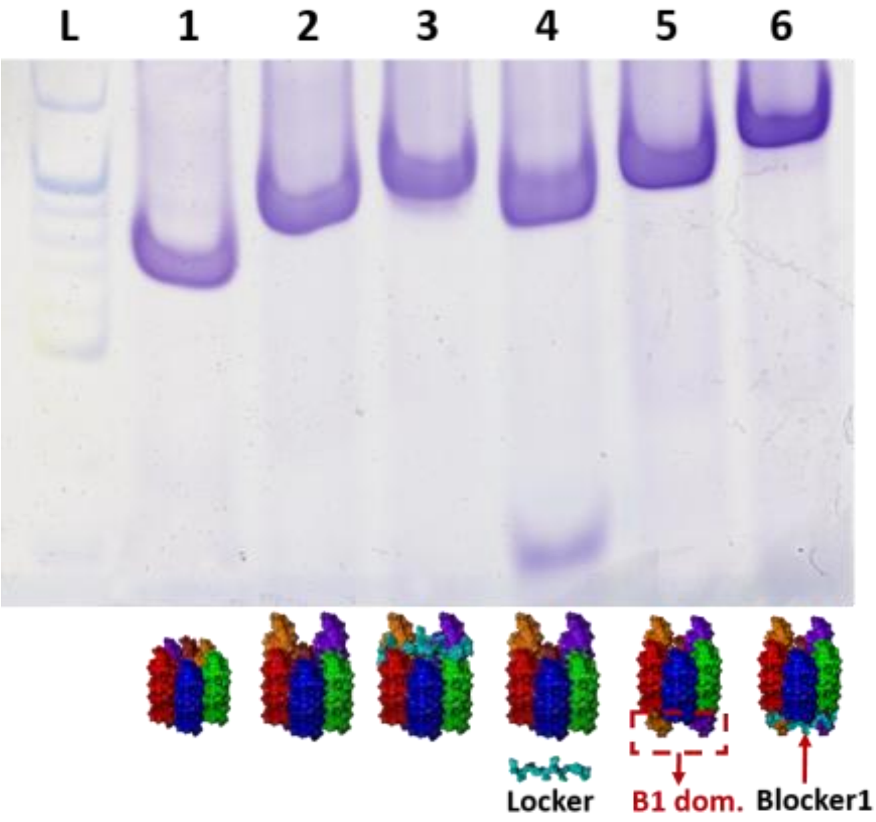

Supplementary Figure 34. PAGE (5%) analysis of 3 μM nanogatekeeper, running at 110 V for 70 min in an ice-water bath. Lane 1: DNA nanochannel. Lane 2: o-DNGK without bottom blocker

docking region. Lane 3: nanostructure in lane 2 is plugged by locker. Lane 4: nanostructure in lane 3 with addition of 3 mM ATP. Lane 5: o-DNGK with bottom blocker docking region (red dash box). Lane 6: a blocker (termed blocker 1) plugs the nanostructure in lane 5, forming closed state nanogatekeeper. Lane L: 20 bp DNA ladder.

7. Transport of biomolecules into cell-mimicking vesicles

Following construction of membrane-spanning nanogatekeeper and the signaling network, a key challenge arises: transport of reaction substrates into the chamber of the membrane compartment. To address this challenge, we investigated the transport efficiency of different molecules with incubation and electroporation.

7a. Purification of fluorescent dye-modified restriction endonuclease

To address the challenge of transporting biochemical reaction reagents across the biomimicking membrane, we studied transport efficiency with two methods, including incubation and electroporation. Since electroporation produced better results for protein enzymes, it was adopted for AMSsys construction.

Initially, the DNA stands were modified with a fluorescent dye. To covalently modify the restriction endonuclease with fluorophore, the powder of sulfo-Cy5 was added into 20 μL aqueous Pvu II with a pipette tip and left to react overnight, followed by centrifugation of the mixture at 14,000 rpm for 10 min in a 3 kd ultrafiltration device and washing twice. The intercepted volume could be used for two experiments and the rest was stored at -20 °C.

7b. Transport of reagents with incubation

As reported in our previous work, nucleic acids can be readily transferred into our giant vesicles. Initially, we studied the transport efficiency of different samples using a humidified incubator at 37 °C with an atmosphere of 5–7% CO_2 for 48 h. A 400 μL solution of giant membrane vesicles was mixed with DNA samples (E, M and F), respectively, and imaged and analyzed with the OLYMPUS FV1000 confocal laser scanning microscope. For fluorescence study, an 800 μL incubated sample was centrifuged for 3 min, and a 200 μL sample located in the middle-bottom layer was extracted for analysis.

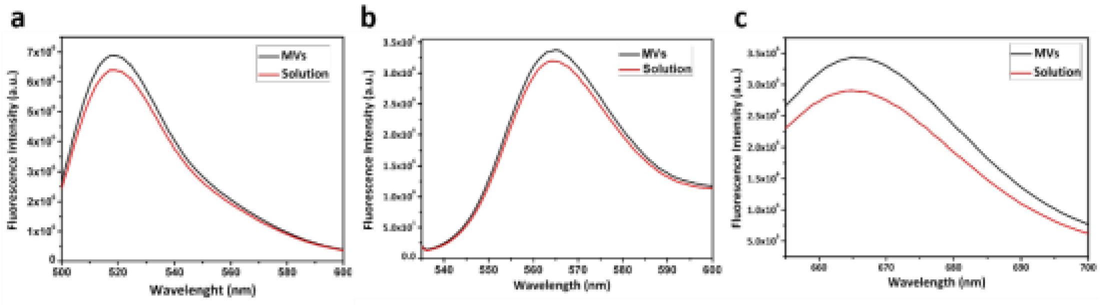

Supplementary Figure 35. Fluorescence intensity change of 0.5 μmole DNA samples (E strand, M strand and F strand) incubated with 800 μL giant membrane vesicles for 48 h and then centrifuged at 1,600 rpm for 3 min. Detection volume=200 μL, λex= 492 nm; 525 nm; 646 nm, respectively; bandpass=5 nm.

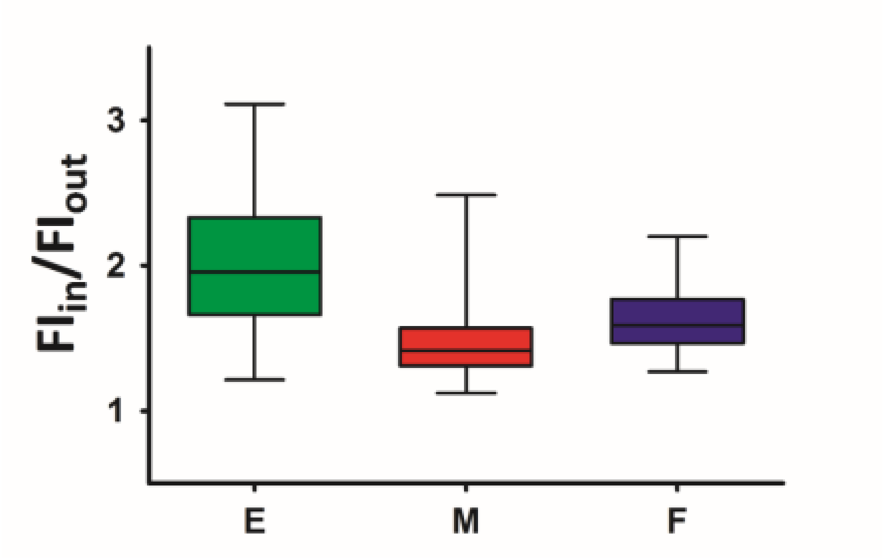

Supplementary Figure 36. Statistical fluorescence intensity ratio of vesicular chamber to

environment (FI_{in} / FI_{out}) for each 500 nM DNA sample. E, M and F were incubated with 400 μM giant vesicles, respectively, and then incubated for 48 h at 37 °C. Each column represents the collected sample population of at least 145 giant membrane vesicles.

7c. Transport of reagents with electroporation

All electrophoresis experiments were performed on the Gene Pulser Xcell™ electrophoresis system (Bio-Rad). Electroporation cuvettes and microcentrifuge tubes were pre-chilled on ice. The samples of signaling network were thoroughly mixed with a 400 μL solution of giant membrane vesicles and 100 μL electroporation medium, and then they were electroporated into the chamber of giant membrane vesicles by exponential decay with 300 V, 500 μF in an ice-water bath. Afterwards, 400 μL were extracted into a 15-mm dish and incubated at 37 °C for 10 min. Finally, chol-c-DNGK was added and incubated for 30 min (for all AMSsys construction). The fluorescence intensities were analyzed with the Olympus FV1000 confocal laser scanning microscope and then GraphPad Prism 5.

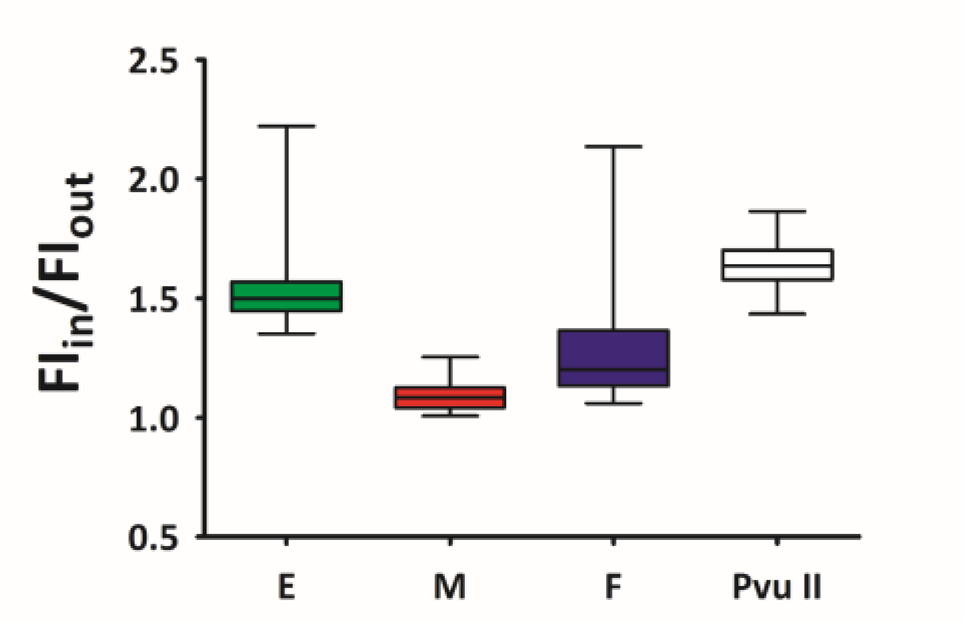

Supplementary Figure 37. Statistical fluorescence intensity ratio of vesicular chamber to environment (FI_{in}/FI_{out}) for each 600 nM sample. Each sample was incubated with 500 μL giant vesicles containing 100 μL of electroporation buffer. Each column represents the statistical sample population of at least 45 giant membrane vesicles.

8. Regulation of AMSsys without feedback reaction

Preparation of functional giant membrane vesicles

The materials of the signaling network were electroporated into the chamber of giant membrane vesicles; then, chol-c-DNGK was anchored into the membrane by incubation (Methods in section 7c). For the measurements, all samples were imaged and measured by Zeiss LSM 880 confocal laser scanning microscope with an objective lens (63×, Plan-Apochromat N.A. 1.40) with 5.3% (488 channel) or 5.3% (633 channel) laser intensity for AMSsys studies.

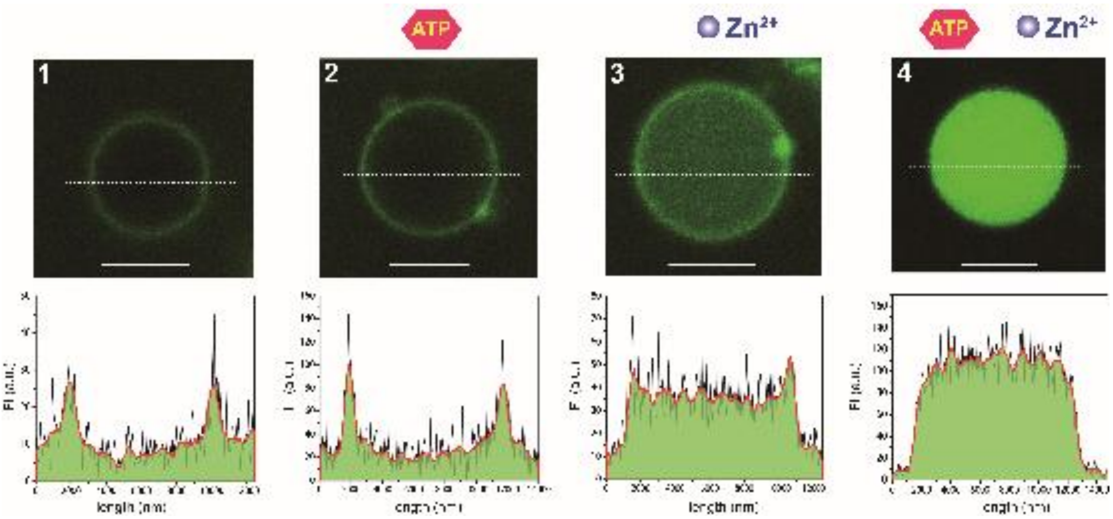

Supplementary Figure 38. Confocal laser scanning microscopy imaging of mimetic cascade reaction controlled by anchored nanogatekeeper in different conditions: Row 1: without ATP and zinc ion. Row 2: addition of 3 mM ATP. Row 3: addition of 0.2 mM zinc ion. Row 4: addition of both ATP and zinc ion. The corresponding picture below denotes the fluorescence intensity of the cross section (white dash line) in each group.

9. Engineering AMSsys in biomimetic GPMVs

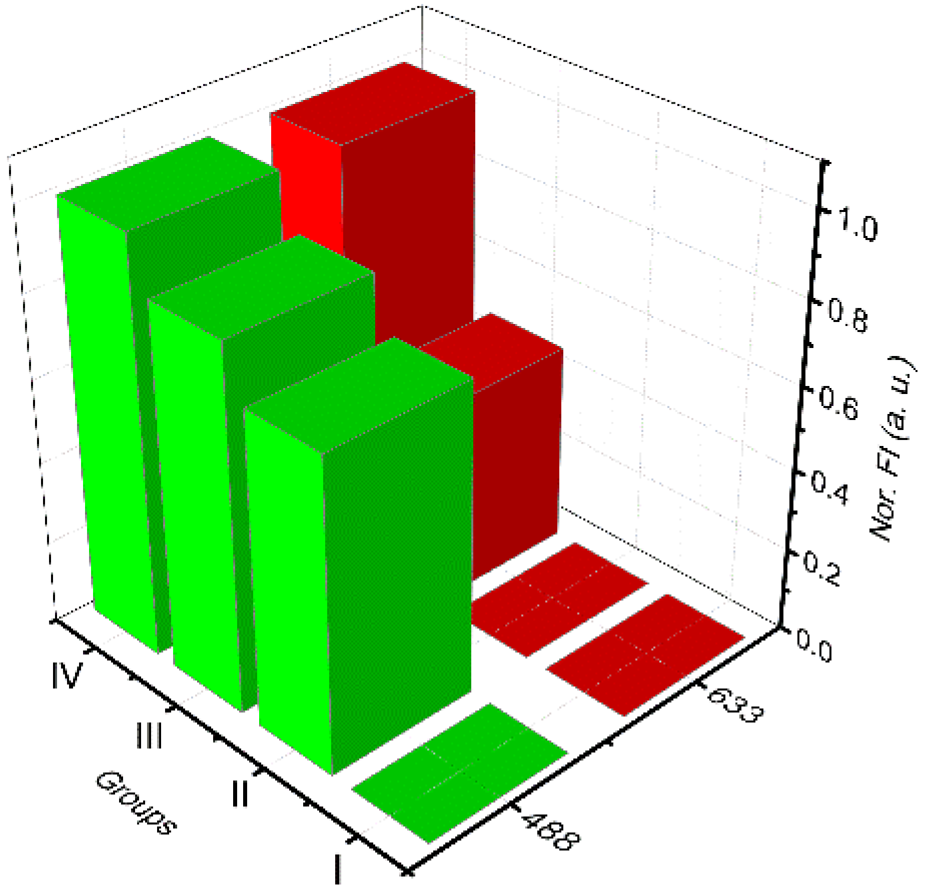

Supplementary Figure 39. Normalized fluorescence intensity correspondence to Figure 5c. Group I: without ATP and ions. Confined signaling network stays inactivated: Cy5-labeled blocker is freely in the chamber, where little 488 signal increases; Group II: Addition of ATP drives the opening of chol-c-DNGK, while signaling network continues to remain inactive by the absence of ion influx triggering. Group III: Addition of only ions (no ATP) cannot trigger the confined reactive network and chol-DNGK remains closed. Group IV: Only in the presence of both ATP and ion influx, can all designed reactions be completed. OriginLab 2016 was used for normalized analysis.

Supplementary Table 8. Sequences of static DNA nanostructure assemblies for above figures.

Fig. / DNA	S5	S6	S10	S12	S13-15	S34	S38	S39
1	✔		✔			✔		
2-short	✔					✔		
3	✔		✔			✔		
4	✔		✔			✔		
5-short	✔					✔		
6	✔		✔			✔		
1-chol		✔		✔	✔		✔	✔
3-chol		✔		✔	✔		✔	✔
4-chol		✔		✔	✔		✔	✔
6-chol		✔		✔	✔		✔	✔
2-ATP	✔	✔		✔	✔	✔	✔	
2Q-ATP			✔					
2-ATP-B1						✔		✔
5-ATP	✔	✔		✔	✔	✔	✔	
5Q-ATP			✔					
5-ATP-B1						✔		✔
lock	✔				✔	✔	✔	✔
FAM-lock			✔					
AF488-lock				✔				
Blocker 1						✔		

✔ denotes that the DNA sequence in left column was used.

Supplementary References

1. M. Langecker, V. Arnaut, T. G. Martin, J. List, S. Renner, M. Mayer, H. Dietz, F. C. Simmel. Synthetic lipid membrane channels formed by designed DNA nanostructures. *Science* **338**, 932-936 (2012). doi: 10.1126/science.1225624;
2. J. R. Burns, A. Seifert, N. Fertig, S. Howorka. A biomimetic DNA-based channel for the ligand-controlled transport of charged molecular cargo across a biological membrane. *Nature Nanotechnol.* **11**, 152 (2016). doi: 10.1038/NNANO.2015.279;
3. S. Krishnan, D. Ziegler, V. Arnaut, T. G. Martin, K. Kapsner, K. Henneberg, A. R. Bausch, H. Dietz, F. C. Simmel. Molecular transport through large-diameter DNA Nanopores. *Nature Commun.* **7**, 12787 (2016). doi: 10.1038/ncomms12787;
4. V. Maingi, J. R. Burns, J. J. Uusitalo, S. Howorka, S. J. Marrink, M. S.P. Sansom. Stability and dynamics of membrane-spanning DNA nanopores. *Nature Commun.* **8**, 14784 (2017). doi: 10.1038/ncomms14784;
5. S. Howorka. Changing of the guard. DNA nanostructures mimic membrane proteins. *Science* **352**, 890-891 (2016). doi: 10.1126/science.aaf5154;
6. J. R. Burns, N. Al-Juffali, S. M. Janes, S. Howorka. Membrane-Spanning DNA Nanopores with Cytotoxic Effect. Angew. Chem. Int. Ed. **53**, 12466–12470 (2014). doi: 10.1002/anie.201405719
7. J. R. Burns, E. Stulz, S. Howorka. Self-Assembled DNA Nanopores That Span Lipid Bilayers. Nano Lett. 13, 2351−2356 (2013). doi: 10.1021/nl304147f
8. X. Zheng et al. Fluorescence Resonance Energy Transfer-Based DNA Nanoprism with a Split Aptamer for Adenosine Triphosphate Sensing in Living Cells. Anal. Chem. 89, 10941-10947 (2017). doi: 10.1021/acs.analchem.7b02763
9. R. Peng et al. Engineering a 3D DNA-Logic Gate Nanomachine for Bispecific Recognition and Computing on Target Cell Surfaces. J. Am. Chem. Soc. 140, 9793−9796 (2018). doi: 10.1021/jacs.8b04319
10. Y. Jiang, N. Liu, W. Guo, F. Xia, L. Jiang. Highly-efficient gating of solid-state nanochannels by DNA supersandwich structure containing ATP aptamers: A nanofluidic IMPLICATION logic device. *J. Am. Chem. Soc.* **134**, 15395−15401 (2012). doi: 10.1021/ja3053333.
11. P. Liu, Y. Zhao, X. Liu, J. Sun, D. Xu, Y. Li, Q. Li, L. Wang, S. Yang, C. Fan, J. Lin. Charge

neutralization drives the shape reconfiguration of DNA nanotubes. Angew. Chem. Int. Ed. **57**, 5418–5422 (2018). doi: 10.1002/anie.201801498.

12. J. R. Burns, S. Howorka. Defined Bilayer Interactions of DNA Nanopores Revealed with a Nuclease-Based Nanoprobe Strategy. ACS Nano, 12, 3263–3271 (2018). doi: 10.1021/acsnano.7b07835.
13. D. Han, C. Wu, M. You, T. Zhang, S. Wan, T. Chen, L. Qiu, Z. Zheng, H. Liang, W. Tan. A cascade reaction network mimicking the basic functional steps of adaptive immune response. *Nature Chem.* **7**, 835–841 (2015). doi:10.1038/nchem.2325;
14. D. Y. Zhang, A. J. Turberfield, B. Yurke, E. Winfree. Engineering entropy-driven reactions and networks catalyzed by DNA. *Science* **318**, 1121-1125 (2007). doi: 10.1126/science.1148532;
15. L. He, D. Lu, H. Liang, S. Xie, X. Zhang, Q. Liu, Q. Yuan, W. Tan. mRNA-Initiated, Three-dimensional DNA amplifier able to function inside living cells. *J. Am. Chem. Soc.* **140**, 258–263 (2018). doi: 10.1021/jacs.7b09789;
16. Y. Lv, L. Cui, R. Peng, Z. Zhao, L. Qiu, H. Chen, C. Jin, X. B. Zhang, Weihong Tan. Entropy beacon: A hairpin-free DNA amplification strategy for efficient detection of nucleic acids. *Anal. Chem.* **87**, 11714−11720 (2015). doi: 10.1021/acs.analchem.5b02654;
17. R. Peng, H. Wang, Y. Lyu, L. Xu, H. Liu, H. Kuai, Q. Liu, Weihong Tan. Facile assembly/disassembly of DNA nanostructures anchored on cell-mimicking giant vesicles. *J. Am. Chem. Soc.* **139**, 12410–12413 (2017). doi: 10.1021/jacs.7b07485;
18. J. Li, W. Zheng, A. H. Kwon, Y. Lu. In vitro selection and characterization of a highly efficient Zn (II)-dependent RNA-cleaving deoxyribozyme. *Nucleic Acids Res*. **28**, 481–488 (2000). doi: 10.1093/nar/28.2.481;
19. M. You, Y. Lyu, D. Han, L. Qiu, Q. Liu, T. Chen, C. S. Wu, L. Peng, L. Zhang, G. Bao, W. Tan. DNA probes for monitoring dynamic and transient molecular encounters on live cell membranes. *Nature Nanotechnol.* **12**, 453–459 (2017). doi:10.1038/nnano.2017.23;
20. W. Tang, H. Wang, D. Wang, Y. Zhao, N. Li, F. Liu. DNA tetraplexes-based toehold activation for controllable DNA strand displacement reactions. *J. Am. Chem. Soc.*, **135**, 13628–13631 (2013). doi: 10.1021/ja406053b;
21. Y. Chen, Y. Song, F. Wu, W. Liu, B. Fu, B. Feng and X. Zhou. A DNA logic gate based on strand displacement reaction and rolling circle amplification, responding to multiple low-

abundance DNA fragment input signals, and its application in detecting miRNAs. *Chem. Commun.* **51**, 6980 (2015). doi: 10.1039/c5cc01389e;

22. Huang J. et al. Competition-mediated pyrene-switching aptasensor: probing lysozyme in human serum with a monomer-excimer fluorescence switch. *Anal. Chem.* **82**, 10158–10163 (2010). doi: 10.1021/ac102277p
